# Bridging Experiment and Theory: Insights into the Photoinitiation Efficiency of Acrylate and Methacrylate Derivatives of 2-Mercaptobenzothiazole in Radical Photopolymerization of Selected Acrylates

**DOI:** 10.3390/polym18212613

**Published:** 2026-10-22

**Authors:** Agnieszka Skotnicka, Maciej Witwicki, Jarosław J. Panek, Aneta Jezierska, Janina Kabatc-Borcz, Adrian A. Chelewski

**Affiliations:** 1 Faculty of Chemical Technology and Engineering, Bydgoszcz University of Science and Technology, Seminaryjna 3, 85-326 Bydgoszcz, Poland; janina.kabatc@pbs.edu.pl; 2 Faculty of Chemistry, University of Wrocław, ul. F. Joliot-Curie 14, 50-383 Wrocław, Poland; maciej.witwicki@uwr.edu.pl (M.W.); jaroslaw.panek@uwr.edu.pl (J.J.P.); aneta.jezierska@uwr.edu.pl (A.J.); 3 Independent Researcher, 85-326 Bydgoszcz, Poland; chelewskiadrian@gmail.com

**Keywords:** acrylate derivatives, methacrylate derivatives, 2-mercaptobenzothiazole, initiators, photopolymerization, DFT, EPR spectroscopy, kinetics

## Abstract

Novel copolymerizable one-component Type I photoinitiators based on 2-mercaptobenzothiazole were considered for initiation of radical polymerization of acrylate monomers. For this purpose, ten diversely substituted compounds were taken into consideration. Based on quantum-chemical calculations in both the ground and excited electronic states, the possible mechanism of generation of initiating radicals was proposed. It was found that S–C bond cleavage led to the formation of thiyl (RS^•^) and carbon-centered radicals (R^•^), and was the preferred pathway for all initiators studied. Time-dependent DFT simulations showed that for all considered compounds, homolytic bond dissociation was feasible. The results obtained from the EPR spectra recorded during in situ irradiation of the photoinitiator revealed that the in silico predicted dissociation pathway is consistent with the experimental observations. The photoinitiation ability of new 2-mercaptobenzothiazole-based compounds was subsequently evaluated through kinetic studies of the photopolymerization of selected acrylate monomers. Their practical performance was further benchmarked against the commercial photoinitiators Irgacure 819 (BAPO) and Irgacure 184 under identical irradiation conditions, demonstrating that several synthesized derivatives achieved comparable or higher final monomer conversions depending on the monomer structure. Among the investigated systems, two acrylate and two methacrylate derivatives exhibited the most promising polymerization performance, with the highest efficiency observed for the system based on 2-hydroxyethyl acrylate (HEA). Correlations between experimental photopolymerization parameters and computational descriptors are semi-quantitative, and—depending on the monomer—highlight the significance of S_1_ excitation energy, S–C bond dissociation energy and spin populations in the thiyl and thiazole moieties.

## 1. Introduction

Photopolymerization is a process in which monomers are transformed into a cross-linked polymer through a chain reaction initiated by reactive species, such as free radicals or ions, which are generated upon exposure to visible or ultraviolet (UV) light [1]. The primary benefit of utilizing UV radiation to initiate such reactions is the attainment of remarkably elevated polymerization rates [2]. Consequently, the transformation from a liquid formulation (initial composition) to a solid polymerized product occurs within fractions of a second. Radiation-curing technologies offer a wide range of economic advantages when compared with conventional thermal processes [3,4]. These systems are distinguished by their low energy demand and the capacity to facilitate reactions at ambient temperature. Furthermore, they employ non-polluting and solvent-free formulations, permit the selective curing of specific areas within a given component, and facilitate the fabrication of materials with tailored properties [5,6]. The aforementioned advantages have led to the widespread use of radiation curing in many fields of science, medicine, and industry. In medicine, its applications include wound dressing materials and drug microencapsulation techniques [7,8,9]. In the domain of dentistry, it remains a fundamental component of dental fillings [10,11]. Furthermore, the application of radiation curing has emerged as a pivotal element in the development of hard and soft contact lenses, as well as in the fabrication of scaffolds for tissue engineering applications. In the technological and industrial sectors, the design of UV-curable varnishes and paints, functional coatings with tailored properties, adhesives, and materials used in graphics is contingent upon this technology [12,13,14]. This approach has found widespread application in various fields, including microelectronics, microlithography, 3D printing, machining processes, photolithography, and the production of optoelectronic components [15].

A key component of photopolymerization systems is the photoinitiator. This compound is able to generate reactive species that initiate polymerization upon exposure to UV or visible light [16,17,18]. According to the prevailing scholarly consensus, photoinitiators are generally classified into two categories: Type I, also known as cleavage-type, and Type II, which is classified as hydrogen-abstraction- or electron-transfer-type [19]. Type I photoinitiators, also known as one-component or cleavage photoinitiators, undergo direct photodissociation after absorbing a photon. The homolytic bond cleavage results in the formation of free radicals, which possess the capacity to initiate polymerization [20]. Conversely, Type II photoinitiators need the presence of a co-initiator, for example, an amine. Upon light absorption, the excited photoinitiator interacts with the co-initiator, generating radicals through hydrogen abstraction or electron transfer. This two-component mechanism contributes to the increased complexity of Type II systems, while concurrently enabling enhanced flexibility and tunability of reactivity through the selection of suitable co-initiators [21].

**Scheme 1:**
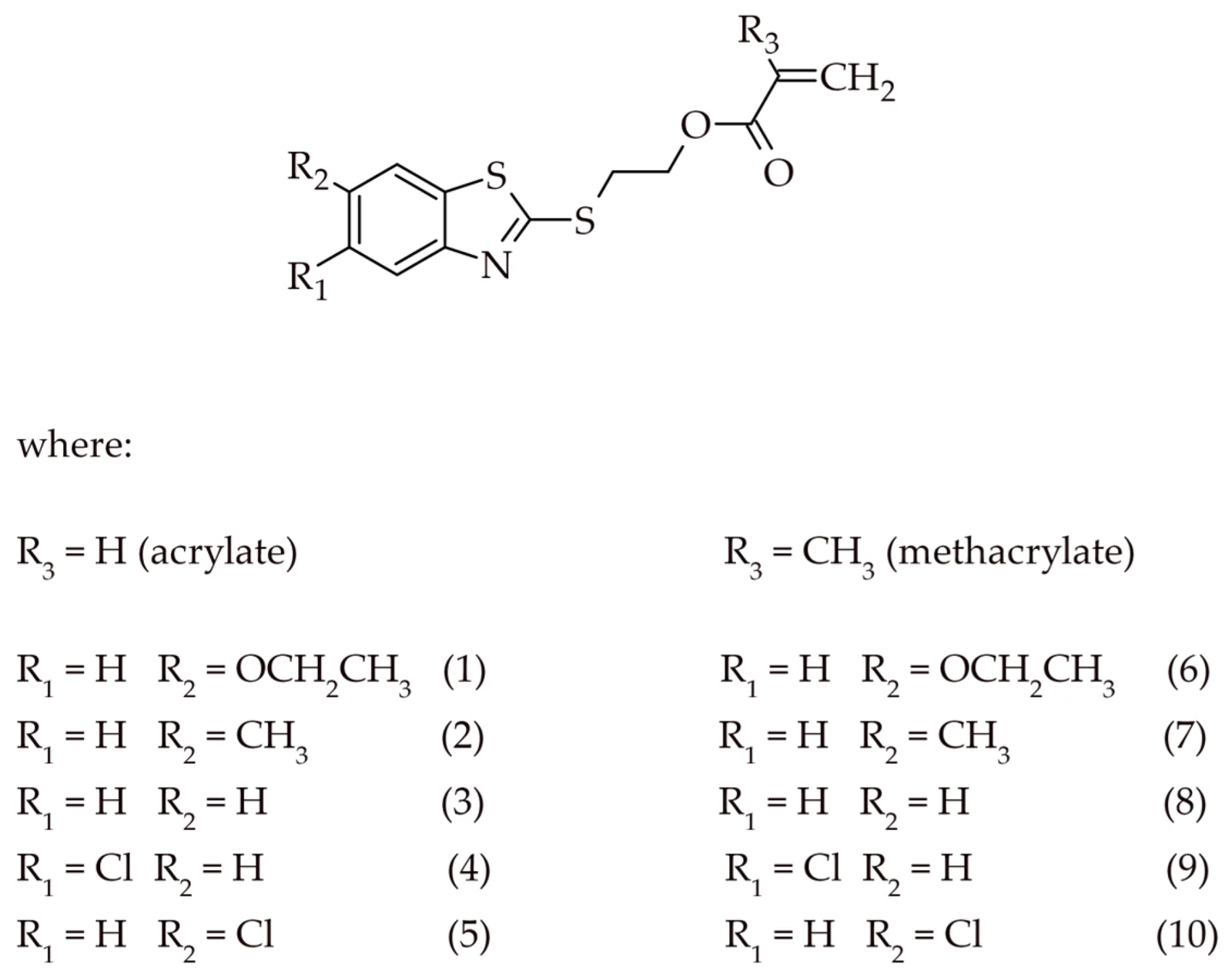
Structures of acrylate (1–5) and methacrylate (6–10) derivatives of 2-mercaptobenzothiazole.

In this study, acrylate and methacrylate derivatives of 2-mercaptobenzothiazole (1–10) (Scheme 1), previously synthesized and structurally characterized in our earlier studies [22,23], and in related prior work [24], were investigated as potential components of photoinitiating systems for the radical polymerization of selected acrylates. During UV irradiation, the intermolecular 2-mercaptobenzothiazole H-abstractors are excited and react with neighboring C−H bonds of the monomer or polymer side chains, leading to the formation of UV-crosslinked acrylic polymers. The present work focuses on elucidating the radical-generation pathways of these compounds and evaluating their photoinitiating performance through a combined experimental and theoretical approach.

The investigated compounds contain a 2-mercaptobenzothiazole moiety and an acryloyloxy-ethyl or methacryloyloxy-ethyl group in their structure. The presence of this polymerizable unsaturated ester group designates these compounds as copolymerizable photoinitiators. Copolymerizable photoinitiators, comprising both Type I and Type II, constitute a distinct class of compounds that possess the capacity to initiate polymerization upon light irradiation. Concurrently, these compounds may bond to the evolving polymer network through copolymerization with monomer units, limiting their migration from the polymer [25]. Incorporating such photoinitiators into the polymer matrix significantly reduces their extractability by solvents, thereby minimizing potential toxicity and enhancing long-term stability. Consequently, copolymerizable photoinitiators are especially suited for regulated applications, such as coatings for food contact materials and pharmaceutical packaging [26]. The aforementioned benefits include enhanced chemical resistance, improved mechanical durability of the cured material, and superior control over network formation and crosslinking density [27]. The properties of copolymerizable photoinitiators render them a compelling and sustainable alternative to conventional systems, particularly within the domain of advanced light-curable technologies.

**Scheme 2:**
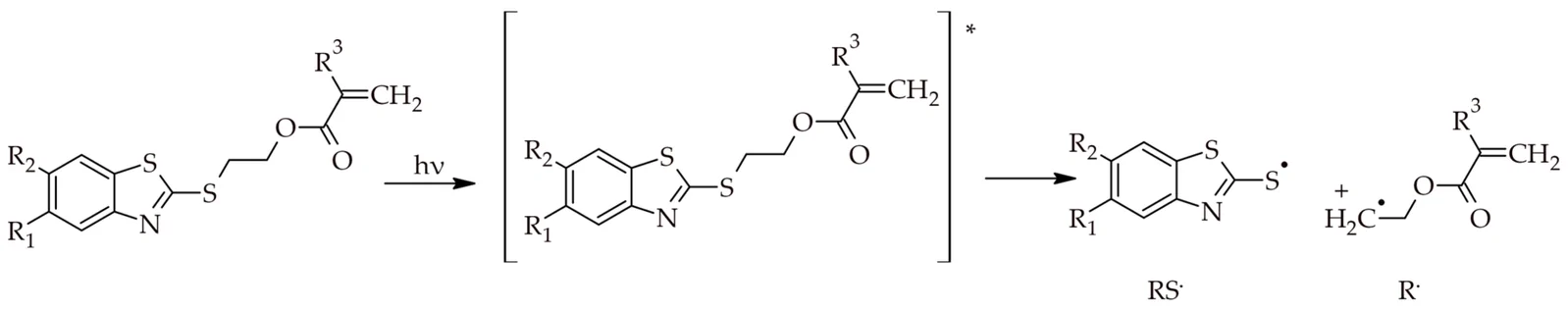
Photoinduced generation of thiyl (RS^•^) and carbon-centered radicals (R^•^). The asterisk (*) indicates an excited state.

As shown by the combined theoretical and experimental results presented herein, the investigated compounds exhibit Type I-like photocleavage behavior involving homolytic S–C bond cleavage (Scheme 1). Upon light absorption, homolytic cleavage of the S–C bond is proposed to generate thiyl (RS^•^) and carbon-centered (R^•^) radicals, as supported by the combined theoretical calculations and EPR measurements discussed in the Section 3 (Scheme 2).

Thiyl radicals based on the 2-mercaptobenzothiazole structure can be generated by quenching the triplet excited states of photosensitizers, such as isopropylthioxanthone (ITX), camphorquinone (CQ), benzophenone (BP), and benzoyl peroxide (BPO), in the presence of 2-mercaptobenzothiazole (MBT) [28,29,30]. This process is illustrated in Reaction 1:

(1) $$\mathrm{PI}\overset{h\nu }{\to }PI^{*}+\mathrm{MBT}\to RS^{\cdot }+\mathrm{PI}H^{\cdot }$$

This reaction is facilitated by the relatively low bond dissociation energy (BDE) of the S–H bond in MBT (83.2 kcal/mol [31]; 83.5 kcal/mol [32]) which favors hydrogen abstraction and thiyl radical formation.

As an alternative, thiyl radicals can be efficiently generated via photolysis of a Type I photoinitiator, such as 2,2′-dithiobisbenzothiazole (BTDS). When exposed to light, BTDS and similar disulfide compounds typically undergo cleavage of the S–S bond, resulting in the formation of two sulfur-centered radicals (RS^•^) (Reaction 2) [30,31]. According to the literature, this dissociation process can occur on an ultrafast timescale of less than 10 picoseconds [31].

(2) $$\mathrm{BTDS}\overset{\mathrm{h\nu}}{\to }{\mathrm{BTDS}}^{*}\to 2{\mathrm{RS}}^{\cdot }$$

A third potential pathway for thiyl radical formation involves energy transfer between a photoexcited photoinitiator and a disulfide compound. According to literature data on the triplet-state energy levels of disulfides, some studies suggest that, upon light absorption, isopropylthioxanthone (ITX) undergoes intersystem crossing to its triplet state (^3^ITX). This triplet state can then efficiently transfer energy to disulfides such as BTDS. This process results in cleavage of the S–S bond and formation of two sulfur-centered radicals (thiyl radicals). The high quenching rate constant observed for the interaction between the ^3^ITX and disulfides supports the efficiency of this mechanism, indicating that the energy transfer and subsequent radical generation occur on a rapid timescale [30]. The overall process is illustrated in Reaction 3:

^3^ITX + BTDS → ITX + ^3^BTDS → ITX + 2RS^∙^ (3)

Sulfur-centered radicals (RS^•^) are effective initiating species in conventional radical photopolymerization processes [16,28,30,31] and thiol-ene systems. These processes are widely used in radiation curing technologies. RS^•^ radicals are attractive due to their low sensitivity to oxygen and high reactivity toward a broad range of carbon–carbon double bonds. The addition of thiyl radicals to double bonds may cause back-fragmentation of the resulting monomer-derived radical. The equilibrium position in this reversible process depends on the thiyl radical and double-bond combination involved [16]. Spectroscopically, thiyl radicals exhibit an absorption band centered at approximately 580 nm in acetonitrile; however, theoretical calculations predict a maximum absorption near 510 nm [31]. This characteristic can be useful for detecting and monitoring these transient species under photoirradiation conditions.

The objective of this study is to address the following research aims:

(1) To evaluate the photoinitiation efficiency of acrylate and methacrylate derivatives of 2-mercaptobenzothiazole in the radical polymerization of selected acrylates;

(2) To identify which radical species are responsible for initiating the polymerization process, both experimentally using electron paramagnetic resonance (EPR) spectroscopy with spin trapping, and theoretically using quantum-mechanical calculations (in other words, the study of the mechanism of primary and secondary photochemical processes);

(3) To determine selected kinetic parameters of the polymerization reactions through the utilization of differential scanning calorimetry (DSC).

In order to fulfill the objectives and make the study comprehensive, experimental investigations as well as Density Functional Theory (DFT) [33,34] simulations were employed.

## 2. Materials and Methods

### 2.1. Experimental Section

Details of the synthesis and spectroscopic properties of the investigated compounds have already been described in Refs. [22,23]. Our next studies were focused on reaction mechanism investigations, based on predictions made by application of quantum-chemical approaches in the electronic ground and excited states. In order to confirm theoretical predictions, the electron paramagnetic resonance (EPR) [35,36] spectroscopic method was applied. Additionally, the kinetic parameters of the polymerization process of selected monomers were determined using the calorimetric method [37,38].

### 2.2. Computational Methods and Procedures

Quantum-chemical simulations based on Density Functional Theory (DFT) [33,34] were performed for the species considered in the fragmentation pathways of acrylate and methacrylate derivatives of 2-mercaptobenzothiazole (1–10). Molecular models of the investigated species, constructed with the GaussView 6.1.1 program [39], were the basis for further simulations. Energy minimization was carried out in the electronic ground state using M06-2X [40] and CAM-B3LYP [41] functionals with the def2-TZVP basis set [42,43]. The gas phase conditions were assumed, without explicit or implicit solvent, as the primary objective of the calculations was to compare the relative energetic parameters within the investigated series rather than to reproduce a particular reaction environment. For each investigated model harmonic frequencies were computed, confirming that the obtained structures corresponded with energy minima on the Potential Energy Surface (PES). Vibrational analysis allowed for the use of thermally corrected energetic parameters for calculation of bond dissociation energies (BDEs): the Supplementary Materials contain the BDEs derived from (a) the electronic energies, (b) the sum of electronic and thermal energies, and (c) the Gibbs free energies; the main text indicates the use of electronic energies or the Gibbs free energies as BDEs depending on the particular case. The vibrational, rotational and translational thermal corrections were calculated at T = 298.15 K and p = 1 atm, within harmonic oscillator and rigid rotor approximations. In the next step, scanning with the relaxation method (i.e., a scan in which the S–C bond coordinate was, at a given scan point, kept fixed at values starting from the optimized value and increasing with 0.2 Å increments, but optimizing all the remaining degrees of freedom) was applied for the S–C bond from photoinitiator 5 to get an energy profile associated with the bond dissociation. The DFT/CAM-B3LYP/def2-TZVP level of theory was used for this purpose. Time-Dependent Density Functional Theory (TD-DFT) [44] was further used to investigate molecular features of acrylate and methacrylate derivatives of 2-mercaptobenzothiazole (1–10) in the electronic excited states. Single-point calculations at the TD-DFT/M06-2X/def2-TZVP and TD-DFT/CAM-B3LYP/def2-TZVP levels of theory (for the geometries derived from the ground state calculations as described above) were applied for this purpose, considering five lowest excited singlet and five triplet states. We focused on vertical excitation energies and S–C bond dissociation energies, together with Mulliken spin populations on selected atoms, in our attempts to search for quantitative correlations between experimental and calculated properties. The quantum-chemical calculations were carried out using the Gaussian 16 Rev. C. 01 suite of programs [45].

### 2.3. Electron Paramagnetic Resonance (EPR)

Electron paramagnetic resonance (EPR) measurements were carried out using a EMX Plus cw-EPR spectrometer (Bruker BioSpin GmbH, Rheinstetten, Germany) operating at approximately 9.7 GHz (X-band). The spectrometer was equipped with a teslameter and a frequency counter. Radical generation was initiated in situ by UV irradiation using a Bruker ER 203UV system with a 100 W mercury lamp, providing a broad spectral range of 200–2000 nm. All spectra were recorded at room temperature in glass capillaries using a solution of photoinitiator 5. Toluene (Avantor Performance Materials Poland S.A., pure grade), used as a solvent in all EPR measurements, was dried for 24 h on 3 Å molecular sieves [46]. The magnetic field modulation amplitude was set to 0.1 or 0.5 mT, while the modulation frequency was set to 100 kHz. Microwave power was maintained at 5 mW throughout all measurements. If needed, 5-fold accumulation of spectra was performed. All EPR spectra were simulated using EasySpin [47,48]. Resonator background signals were carefully subtracted from each spectrum.

Direct detection of the generated radicals was performed using photoinitiator 5 at a concentration of 20 mM. For spin trapping experiments [35,49,50,51,52], *N*-*tert*-butyl-*α*-phenylnitrone (PBN; >99%, Sigma-Aldrich/Merck, KGaA Darmstadt, Germany) was used as the spin trap, given its known efficiency in detecting carbon- and sulfur-centered radicals [35,53,54,55,56,57]. Samples were prepared by dissolving 2 mg of PBN in 100 µL of 20 mM solution of photoinitiator 5, sealed in glass capillaries and UV irradiated in situ. Control samples contained 2 mg of PBN in 100 µL of pure toluene.

To evaluate whether UV irradiation of the investigated photoinitiators could additionally initiate radical polymerization via hydrogen atom transfer or electron transfer mechanisms, further experiments were conducted using 3,5-di-*tert*-butyl-*o*-hydroquinone (H_2_Q; 98%, Sigma-Aldrich/Merck) and 3,5-di-*tert*-butyl-*o*-benzoquinone (Q; 98%, Sigma-Aldrich/Merck). Test samples were prepared by dissolving 5 mg of H_2_Q or Q in 100 µL of 20 mM solution of photoinitiator 5. Control samples were prepared similarly, but in 100 µL of pure toluene.

### 2.4. Kinetics of Polymerization Measurements

Kinetic parameters of the polymerization process of selected monomers—butyl acrylate (BA) (≥99%, contains 10–60 ppm monomethyl ether hydroquinone as inhibitor), 1,6-hexanediol diacrylate (HDDA) (99%, stabilized with 90 ppm hydroquinone), trimethylolpropane triacrylate (TMPTA), 2-hydroxyethyl acrylate (HEA) (96%, contains 200–650 ppm monomethyl ether hydroquinone as inhibitor) and pentaerythritol triacrylate (PETA) (stabilized with 300–400 ppm 4-methoxyphenol) initiated by both acrylic and methacrylic derivatives of 2-mercaptobenzothiazole (1–10)—were determined using a TA Instruments DSC Q2000 (TA Instruments, Warsaw, Poland) differential scanning calorimeter equipped with a photometric attachment with a 200 W high-pressure mercury lamp from OmniCure S2000 (Excelitas Canada Inc., Mississauga, ON, Canada)). The measurements were conducted under isothermal conditions (25 °C) in a nitrogen atmosphere. The inert gas flow was 50 mL min^−1^. A high-pressure mercury lamp was applied as the light source, which emitted radiation in the range of 300 to 500 nm. The UV–Vis absorption properties of the investigated photoinitiators, including their absorption maxima and molar absorption coefficients, have been reported in our previous studies [22,23]. These data provide relevant information for assessing the compatibility of the photoinitiators’ absorption characteristics with the irradiation range used in the present study. The intensity of the radiation was constant and equal to 50 mW cm^−2^. The measurements were performed at a sampling interval of 0.05 s per point. Samples at a weight of 30 ± 0.1 mg were placed into an open aluminum pan, and then maintained at the specified temperature (25 °C) for 2 min before each measurement run began. The heat emitted during the polymerization reaction was registered as a function of time. Duplicate measurements were performed for each photoinitiatin system.

The polymerization mixtures were composed of appropriate monomers, including BA, HDDA, TMPTA, HEA, or PETA, and the acrylic and methacrylic derivatives of 2-mercaptobenzothiazole (1–10), which served as photoinitiators. The photoinitiator weights were prepared based on weight concentrations in relation to 1 g of the polymerization mixture. In most cases, the photoinitiator was dissolved directly in the monomer shortly before the measurement. For monomers in which the photoinitiator exhibited limited solubility, the photoinitiator was first dissolved in 1-methyl-2-pyrrolidone (0.1 mL), and the monomer was added immediately prior to the polymerization experiment.

The quantity of heat evolved during the photopolymerization process is proportional to the degree of conversion of carbon–carbon double bonds. The apparent double-bond conversion was estimated from the total heat released during the photopolymerization process using the following Equation (4) [58]:

(4) $$C_{\%}=\frac{{∆H}_{t}}{{∆H}_{0}}\cdot \frac{\mathrm{MW}}{n\cdot m}\cdot 100$$

where C_%_ is the apparent double-bond conversion (%), ΔH_t_ the total heat released during polymerization (total area under the curve f(t) = dH/dt) (mJ), ΔH_0_ the theoretical enthalpy of polymerization of one reactive group (for acrylates the value H_0_ = 78.2 kJ/mol [59] was used) (kJ/mol), MW the molecular weight of the monomer (g/mol), n the number of reactive groups (double bonds) in the monomer, and m the weight of the sample (g). Since the investigated photoinitiators are copolymerizable and contain acrylate or methacrylate groups, the measured heat may include a contribution from the polymerization of these groups; therefore, the calculated values represent apparent conversion values and should not be interpreted as the exact conversion of the main monomer alone.

The polymerization rate was determined from the heat flow recorded during the reaction, following the approach reported in the literature [60]. For the present photo-DSC measurements, the relationship was expressed in the following expanded form:

(5) $$R_{p}=\frac{\frac{\mathrm{dH}}{\mathrm{dt}}\cdot \mathrm{MW}\;\cdot 10^{-6}}{{∆H}_{0}\;\cdot n\;\cdot m}$$

where R_p_ is the apparent polymerization rate (s^−1^), dH/dt is the heat flow recorded during the polymerization reaction (mW), MW is the molecular weight of the main monomer (g/mol), n is the number of reactive double bonds in the monomer, ΔH_0_ is the theoretical enthalpy of polymerization of one acrylate reactive group (78.2 kJ/mol [59]), and m is the total mass of the sample used in the photo-DSC measurement (g). The factor 10^−6^ accounts for the conversion of the units used for heat flow and enthalpy.

Based on the maximum polymerization rate R_p(max)_ and the time at which the maximum heat release t_max_ occurs during the polymerization process, the overall initiation ability of the reaction I_p_ can be determined according to Formula (6) [61]:

(6) $$I_{p}=\frac{R_{p(\max)}}{t_{\max}}$$

## 3. Results and Discussion

### 3.1. Identification of Radical Species Responsible for Polymerization Initiation

#### 3.1.1. Computational Elucidation of Radical Formation Pathways

**Figure 1:**
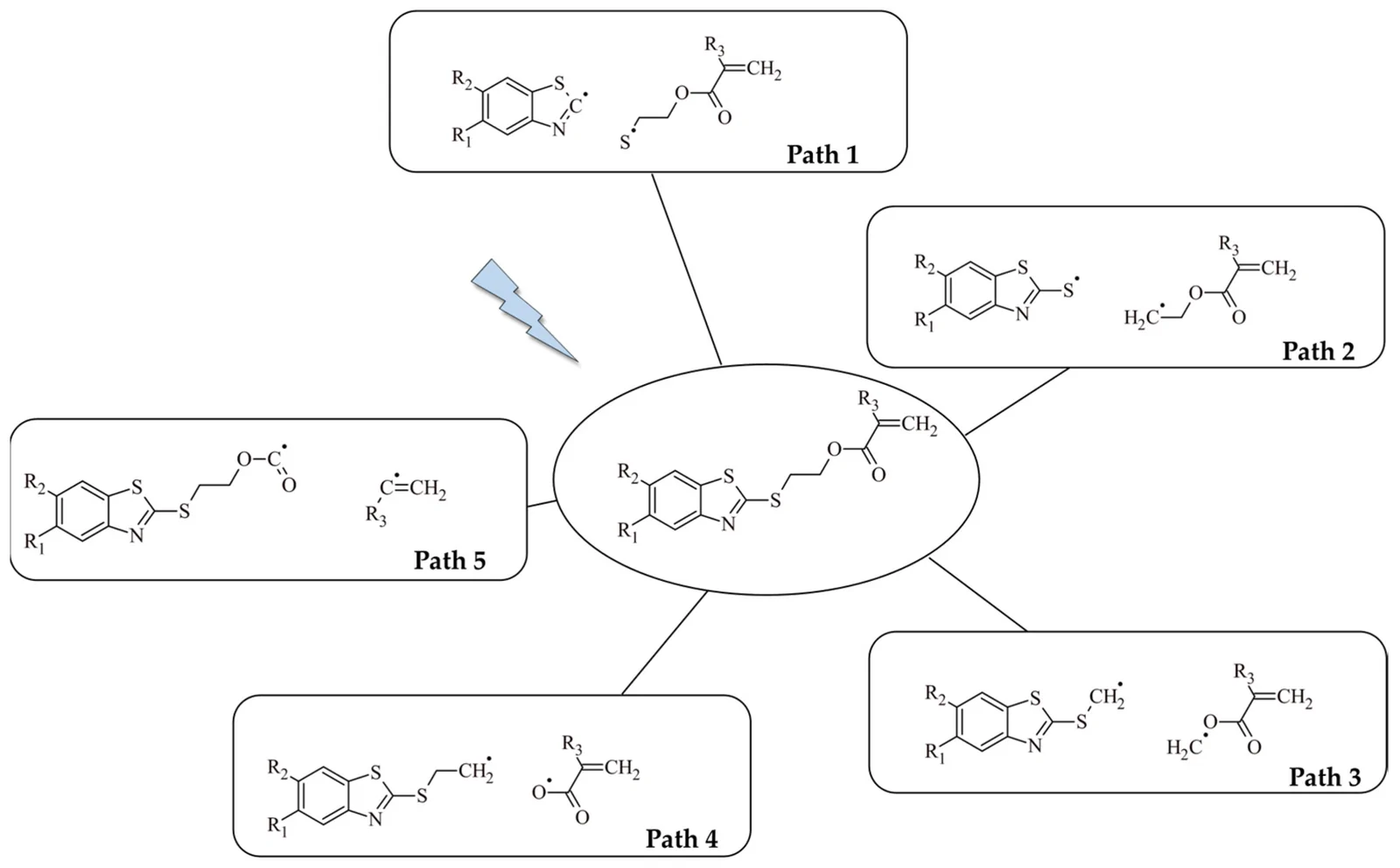
Potential fragmentation pathways of acrylate and methacrylate derivatives of 2-mercaptobenzothiazole (1–10).

An examination of the structure of the acrylate and methacrylate derivatives of 2-mercaptobenzothiazole (1–10) reveals five potential fragmentation pathways, as illustrated in Figure 1.

It has been demonstrated by quantum-mechanical calculations and EPR experiments that the most probable site of fragmentation in derivatives 1–10 is the sulfur-carbon (S–C) bond in the aliphatic chain. The cleavage of this bond generates a thiyl radical (RS^•^) and a carbon-centered radical (R^•^) (Scheme 2).

**Figure 2:**
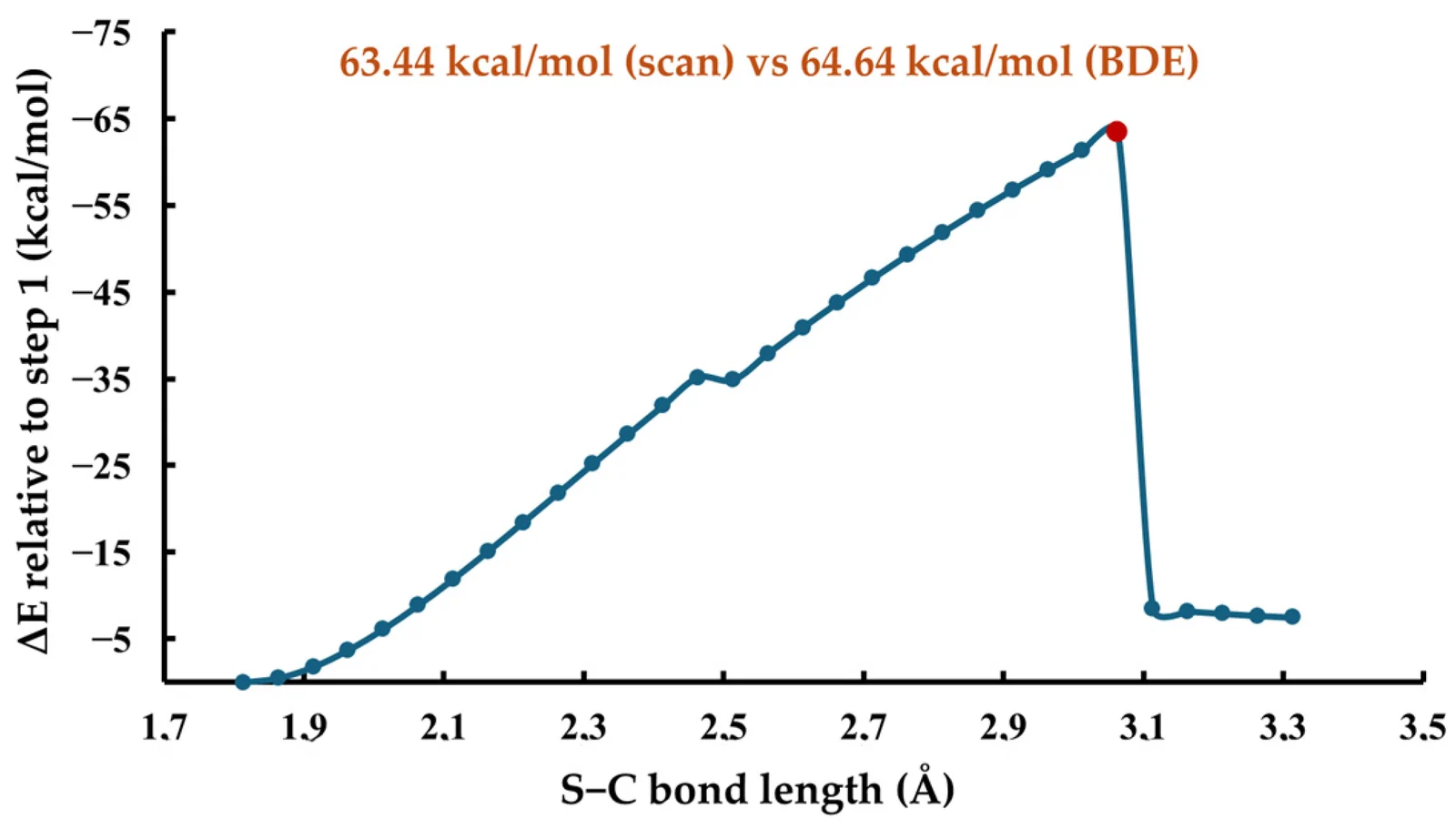
Relative energy changes as a function of the S–C bond length in photoinitiator 5 (CAM-B3LYP/def2-TZVP). BDE—electronic (non-thermal) bond dissociation energy.

Geometry optimization of these derivatives in the ground electronic state further supports this finding: the S–C bond in the aliphatic chain is consistently the longest bond in the molecule. The results derived from both M06-2X/def2-TZVP and CAM-B3LYP/def2-TZVP applied levels of theory predict the S–C bond length to be approximately 1.813 Å, while the C–S–C angle amounts to 101° and 102°, respectively. Other bonds in the aromatic ring and substituents remain within expected ranges, indicating that the molecular backbone is largely rigid. A relaxed scan of the S–C bond provides further evidence for its susceptibility to homolytic cleavage. As the bond length increases from the equilibrium geometry, the energy rises monotonically, consistent with progressive weakening of the S–C bond. A characteristic decrease in the calculated energy is observed around distances corresponding to the bond dissociation energy. This decrease indicates the onset of bond rupture and the formation of radical species. This behavior serves to confirm that the elongation of the S–C bond follows the expected homolytic pathway and is in line with the independent estimates of bond dissociation energies. This tendency is demonstrated in Figure 2, which presents the changes in relative energy as a function of the S–C bond length. The curve demonstrates a gradual increase, followed by stabilization around the energy that corresponds to the calculated bond dissociation energy (at this point the BDE value is derived from electronic energies not corrected for the thermal effects, in agreement with the methodology of the scan). This finding is consistent with homolytic bond rupture.

In addition to the bond length analysis and potential energy scan, the relative stabilities of the radicals formed upon fragmentation were evaluated. Single-point energy calculations performed at the M06-2X/def2-TZVP and CAM-B3LYP/def2-TZVP levels of theory consistently confirmed that the lowest-energy dissociation pathway corresponds to path 2 (Figure 1), involving cleavage of the S–C bond in the aliphatic chain. The energies of the radicals generated in alternative fragmentation routes were significantly higher, rendering these paths less favorable under the studied conditions. The results of these calculations are summarized in Table 1, where both computational methods yield consistent trends, further supporting the assignment of the S–C bond scission as the dominant homolytic cleavage mechanism in derivatives 1–10.

The initiation of the polymerization process occurs in the presence of the reactive species generated upon the absorption of a photon by the acrylate and methacrylate derivatives of 2-mercaptobenzothiazole (1–10). The results of quantum-mechanical calculations indicate that following the absorption of excitation energy, the studied compounds transition from the singlet ground state (S_0_) to the first excited singlet state (S_1_), subsequently undergoing intersystem crossing (ISC) to the triplet state (T_1_). In this triplet state, homolytic bond dissociation becomes feasible, provided that the energy of T_1_ exceeds or is comparable to the bond dissociation energy (BDE) of the targeted bond. As indicated in the literature, according to established photochemical criteria, for dissociation to be effective, the following conditions must be met, among others: (i) the molecule must possess a bond with a relatively low BDE (yet sufficiently high to prevent thermal decomposition in the ground state), and (ii) the energy of the excited state from which dissociation occurs must be equal to or greater than the BDE of that bond [16]. The obtained theoretical results confirm that in all cases, the dissociation energy of the S–C bond is lower than the energy of the triplet state (T_1_), as shown in Table 2. In particular, this table uses electronic BDEs, while thermally corrected BDEs are even lower (see Table S2).

The results of EPR investigations described in the subsequent section of this work suggest the possibility of an alternative initiation pathway in the radical photopolymerization of selected acrylates. In particular, experiments performed in the presence of 3,5-di-*tert*-butyl-*o*-hydroquinone (H_2_Q) indicate that the thiyl radicals generated from the investigated 2-mercaptobenzothiazole derivatives can abstract hydrogen atoms, thereby leading to the formation of new radical species. This behavior is in accordance with the literature, which describes thiyl radicals as being highly reactive intermediates in hydrogen abstraction reactions. As demonstrated by Andrzejewska et al., hydrogen transfer processes involving sulfur-centered radicals can efficiently compete with other radical recombination and abstraction pathways [28]. In addition, studies conducted by Schöneich and co-workers have unequivocally demonstrated that thiyl radicals efficiently abstract hydrogen atoms from alcohols, particularly from C–H bonds on carbon atoms adjacent to hydroxyl or alkoxy groups [62,63].

The investigated photopolymerization systems were monocomponent formulations consisting exclusively of a monomer and a photoinitiator, without the addition of external hydrogen donors such as amines, thiols, benzoxazines, aldehydes, alcohols, or tetrahydrofuran. Under these conditions, the monomer itself might act as a hydrogen donor. To assess this possibility, bond dissociation energies (BDEs) of all C–H bonds in the tested monomers (BA, HDDA, TMPTA, PETA, and HEA) were calculated.

**Scheme 3:**
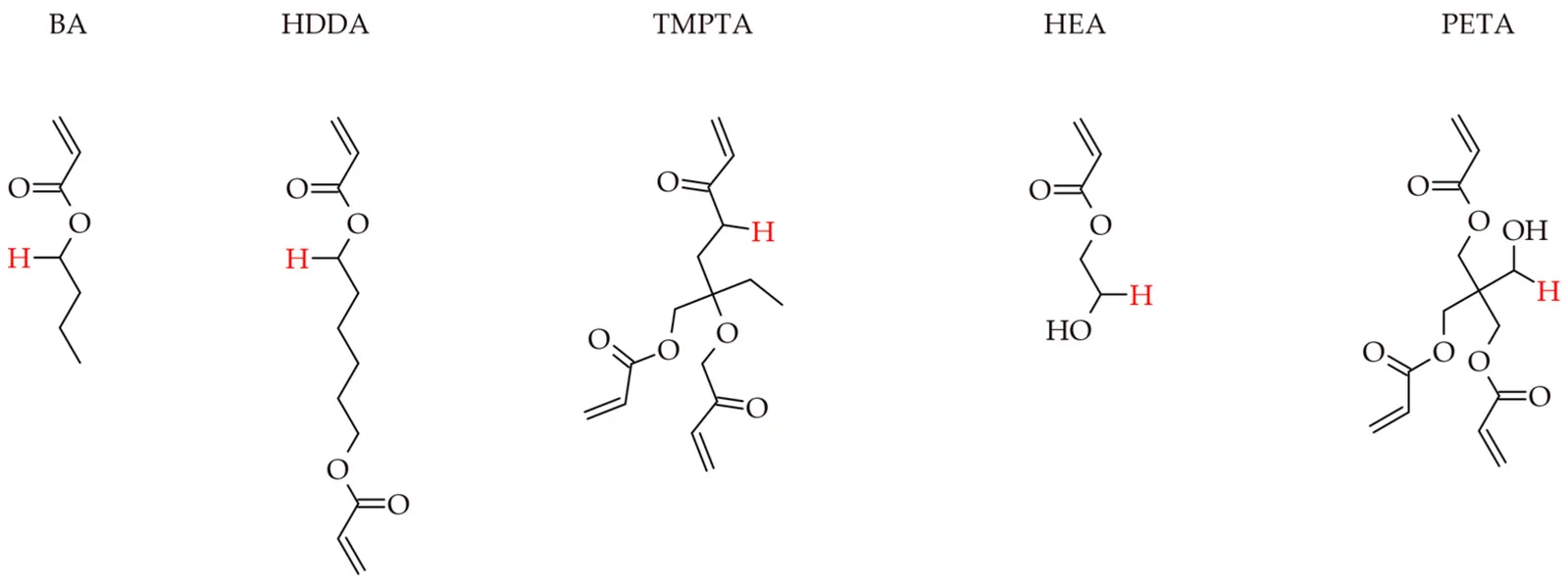
The position of the weakest C–H bond in each monomer.

The computational results clearly indicate which C–H bonds are most susceptible to cleavage and enable the identification of the corresponding “monomer-derived” radical species. In monomers containing hydroxyl groups (PETA and HEA), hydrogen abstraction preferentially occurs from C–H bonds on carbon atoms adjacent to the hydroxyl group. In contrast, in BA, HDDA, and TMPTA, the weakest C–H bonds are associated with carbon atoms adjacent to the ester moiety (Scheme 3).

Nevertheless, the calculated electronic BDE values for all investigated monomers are very similar and do not allow for unambiguous identification of an intrinsically superior hydrogen donor among them (Table 3). Moreover, the predicted BDE values are noticeably higher than those reported for phenolic compounds, which are well known to act as efficient hydrogen atom donors. For example, calculated values at the DFT/B3LYP/6-311++G(3df,2p) level of theory amount to 82.89 kcal/mol for phenol, 72.25 kcal/mol for gallic acid, and 73.72 kcal/mol for epicatechin [64]. Furthermore, experimental studies involving the incorporation of *N*-phenylglycine (NPG) into the HDDA/photoinitiator system did not result in any enhancement of polymerization efficiency. This observation suggests that hydrogen atom transfer (HAT) is not the predominant initiation mechanism in the examined systems. Consequently, even if initiation via monomer-derived alkyl radicals is feasible, its contribution to the overall polymerization efficiency appears to be relatively limited [16].

In consideration of the aforementioned factors, it can be concluded that the observed higher polymerization efficiency in PETA and HEA, in comparison to other monomers, cannot be attributed to potential hydrogen atom transfer alone. This behavior is hypothesized to arise from a combination of specific structural and physicochemical features of the individual monomers.

PETA, as a trifunctional monomer with a distinct molecular architecture, provides a locally favorable spatial arrangement of reactive double bonds, which promotes rapid network formation [65]. In contrast, HEA, being a monofunctional and low-viscosity monomer, benefits from high mobility of both monomer molecules and radical species, enabling efficient propagation with minimal diffusion limitations [66,67]. Furthermore, the presence of hydroxyl groups in both PETA and HEA may facilitate hydrogen bonding and specific intermolecular interactions, which can stabilize transient radical species and modify the local microenvironment, thereby enhancing photoinitiator decomposition and propagation efficiency [68].

#### 3.1.2. EPR Spectroscopic Analysis of Radical Formation

**Figure 3:**
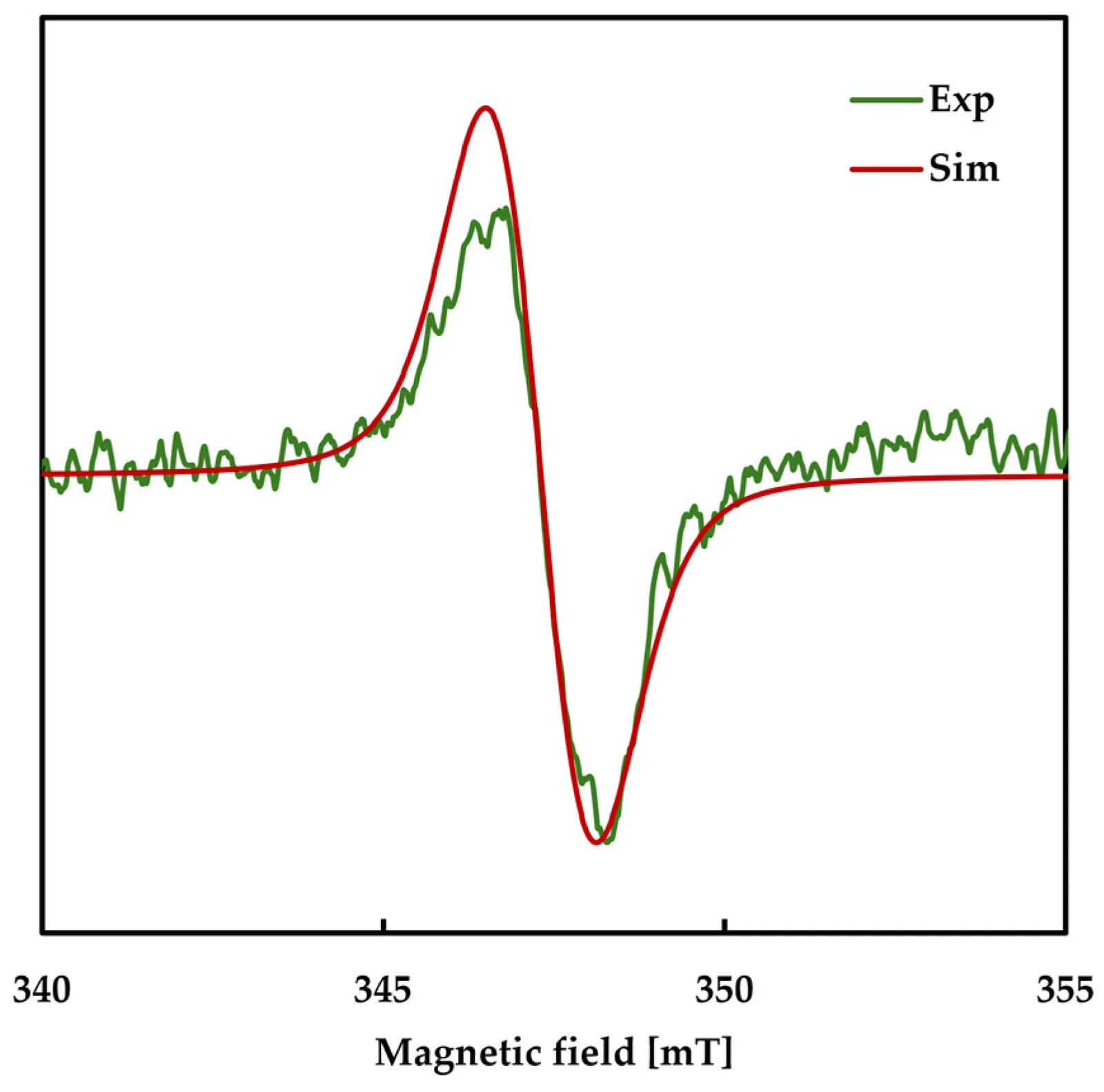
EPR spectrum recorded during in situ irradiation of a 20 mM solution of photoinitiator 5, obtained using a magnetic field modulation amplitude of 0.5 mT and 5-fold signal accumulation.

To experimentally confirm the nature of the radicals generated upon photoexcitation, EPR measurements were performed. Acrylate derivative 5 was selected as a representative case, since it had been identified as one of the most efficient photoinitiators in the series, suggesting a high propensity for radical formation under UV irradiation. The direct detection of radical species was attempted by irradiating a 20 mM solution of photoinitiator 5 in situ. It was only when a relatively high magnetic field modulation amplitude of 0.5 mT was applied, in combination with a five-fold signal accumulation, that a weak but distinct signal was observed after 10 min of irradiation (Figure 3). The spectrum exhibited a single broad line with a peak-to-peak width of approximately 1.5 mT and an isotropic g-value (g_iso_) of 2.0087. This value is consistent with the presence of an organic radical and is higher than typically reported for purely carbon-, nitrogen-, oxygen-, or oxygen–nitrogen-centered species [69].

Since the g value is primarily determined by spin–orbit coupling [69,70,71], the elevated value observed here suggests substantial localization of spin density on a heavier element—most likely sulfur, given the molecular structure of derivative 5. In contrast to alkoxyl radicals (RO^•^), thiyl radicals (RS^•^) possess nearly degenerate *π* orbitals that contribute to significant orbital angular momentum. Consequently, they typically exhibit very short spin–spin relaxation times (T_2_), which results in broad EPR signals that are often below the detection threshold in liquid solution [72,73,74].

However, when sulfur is substituted with a *π*-conjugated system such as CH_2_=CH– or C_6_H_5_–, the *π*-orbital degeneracy is (partially) lifted through delocalization of the unpaired electron across multiple atoms [72,75,76]. In such cases, EPR signals from thiyl radicals can become detectable even in liquid phase. One example is the photochemically generated 2,4,6-tri-(*tert*-butyl)benzenethiyl radical, which exhibits an EPR signal in liquid solution with g_iso_ = 2.0104 and an unresolved hyperfine structure [75,76]. While the g_iso_ value reported for that radical is slightly higher than the one observed in our study, this difference is not unexpected, as the g-tensor (and thus g_iso_) for thiyl radicals is highly sensitive to molecular structure. Another example is the phenylthiyl radical (Ph–S^•^), observed in UV-irradiated diphenyl disulfide, which exhibits a g_iso_ of 2.008 [calculated as g_iso_ = (g_∥_ + 2g_⟂_)/3] closely matching our observation [77].

It is important to note that these g_iso_ values are significantly lower than the typical range of 2.05–2.09 expected for thiyl radicals [78,79] (see also Table 4 in the article of van Gastel et al. [80]). This discrepancy can be attributed to the delocalization of the unpaired electron, which both reduces the angular momentum enabling EPR detection in solution and lowers the g-value [78].

Altogether, the spectrum shown in Figure 3 supports the hypothesis that UV irradiation induces homolytic cleavage of the S–C bond between the aliphatic carbon atom and the sulfur atom (Scheme 2), leading to the formation of a thiyl radical. In this case, the potential delocalization of the unpaired electron over adjacent aromatic systems allows for its detection in liquid phase by EPR and accounts for the relatively low g_iso_ observed. The cleavage of the same bond also generates a carbon-centered radical; however, such species were not observed in the direct measurements, most likely due to their short lifetimes in solution. To address this limitation and to further confirm the formation of thiyl radicals, spin-trapping experiments were employed, a technique particularly well suited for identifying transient radical species of both types [35,53,54,55,56,57].

**Figure 4:**
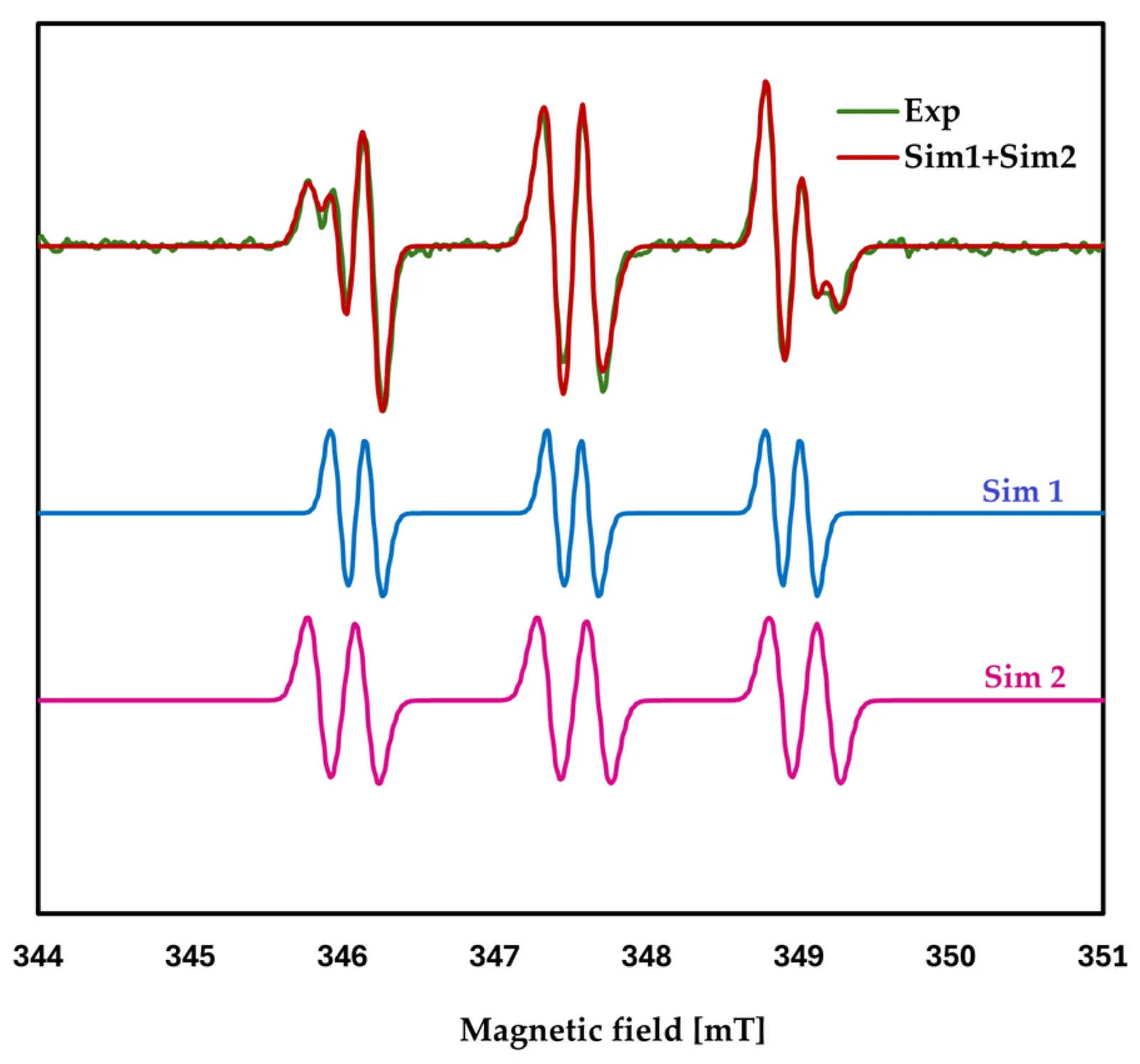
Experimental and simulated EPR spectra recorded as a 5-fold accumulation for the in situ UV-irradiated sample containing both PBN and photoinitiator 5. The simulated spectrum was obtained as an equal combination of two signals: the spin adduct of a thiyl radical (Sim1) and the spin adduct of a carbon-centered radical (Sim2). See the main text for details.

In spin-trapping experiments, a spin trap, in this case PBN, undergoes an addition reaction with short-lived radical species to form a more stable nitroxide radical (spin adduct). This adduct can be detected by EPR and its spectrum provides valuable information on the nature of the trapped radical, particularly through hyperfine coupling constants [35,49,50,51,52]. For the spin-trapping experiments, samples were prepared by dissolving 2 mg of PBN in 100 µL of a 20 mM solution of photoinitiator 5 in toluene. Control samples were prepared analogously but using only PBN and pure toluene. No EPR signal was observed for the control sample, while monitored every 4–5 min for 80 min of UV irradiation. n contrast, for the sample containing both PBN and photoinitiator 5, an EPR signal started emerging approximately 10 min after the beginning of UV irradiation, with the signal reaching its maximum intensity around 25 min (Figure S1). A representative spectrum, recorded as a 5-fold accumulation, is shown in Figure 4.

PBN spin adducts typically show EPR spectra consisting of a triplet of doublets, arising from hyperfine coupling to the ^14^N nucleus (I = 1) and a single *β*-hydrogen (^1^H, I = 1/2). However, in the low- and high-field regions of the spectrum in Figure 4, line patterns indicating overlapping signals are observed, suggesting the presence of two different spin adducts with similar g_iso_ values but different hyperfine coupling constants a_iso_. This experimental spectrum was effectively reproduced by simulation using a combination of two components, labeled as Sim1 and Sim2 in Figure 4. The parameters determined from the fit for Sim1 are g_iso_ = 2.00602, a_iso_(^14^N) = 40.0 MHz (1.43 mT), a_iso_(^1^H) = 6.1 MHz (0.22 mT) and w = 0.50, while for Sim2 g_iso_ = 2.00600, they are a_iso_(^14^N) = 42.6 MHz (1.52 mT), a_iso_(^1^H) = 8.8 MHz (0.31 mT) and w = 0.50, where w represents the weights of the components in the final spectrum.

The smaller a_iso_(^1^H) and a_iso_(^14^N) values found for Sim1 are consistent with literature data for PBN spin adducts of thiyl radicals, whereas these parameters obtained for Sim2 are within the range for PBN adducts of carbon-centered radicals [35,53,54,55,56,57,81,82]. Moreover, the simulation indicates that the two types of spin adducts are formed in approximately equal proportions. This observation is consistent with the formation of the two primary radicals in comparable amounts, as expected for the homolytic cleavage of the S–C bond. However, it should be noted that the relative contributions of the spin adducts may also be influenced by differences in their trapping rates and stability. Therefore, the equal proportions of the spin adducts should not be considered a decisive measure of the concentrations of the primary radicals.

It should be noted that thiyl radical adducts can be difficult to distinguish [35,53]. Here, the Sim1 signal was assigned to the PBN adduct of the thiyl radical by comparing the observed hyperfine coupling constants, a_iso_(^14^N) = 40.0 MHz (1.43 mT) and a_iso_(^1^H) = 6.1 MHz (0.22 mT), with the literature values. This included PBN adducts of: C_6_H_5_–S^•^ with a_iso_(^14^N) = 1.39 mT and a_iso_(^1^H) = 0.18 mT [81], *p*-ClC_6_H_4_–S^•^ with a_iso_(^14^N) = 1.38 mT and a_iso_(^1^H) = 0.18 mT [54], *p*-CH_3_O–C_6_H_4_–S^•^ with a_iso_(^14^N) = 1.39 mT and a_iso_(^1^H) = 0.18 mT [54], and the thiyl radical derived from trimethylolpropane *tris*(3-mercaptopropionate) with a_iso_(^14^N) = 1.39 mT and a_iso_(^1^H) = 0.22 mT [82].

An important point here is that all these reference values were obtained in benzene or toluene, which are low-polarity, aprotic solvents. This is highly relevant, as our measurements were also performed in toluene, making these data directly comparable. By contrast, it is well known that the EPR parameters of nitroxide radicals such as PBN spin adducts are strongly affected by solvent polarity and hydrogen bonding [83,84,85,86]. For instance, both effects tend to decrease g_iso_ and increase a_iso_(^14^N). Consequently, the values reported for PBN adducts of thiyl radicals derived from biomolecules like cysteine, homocysteine or glutathione, which are typically in the range of 1.52–1.57 mT for a_iso_(^14^N) and 0.30–0.35 mT for a_iso_(^1^H) [49,53,55,87], cannot be used as reliable references here, since they were determined in highly polar, protic solvents.

In summary, the spin-trapping experiments demonstrate that UV irradiation of photoinitiator 5 leads to the formation of two types of paramagnetic species, namely carbon-centered and thiyl radicals, in comparable proportions. This observation is consistent with the homolytic cleavage of the S–C bond depicted in Scheme 2 and confirms the generation of radical species capable of initiating polymerization reactions. Nevertheless, although homolytic bond dissociation is the primary photochemical process, the subsequent steps by which these species may initiate the polymerization of acrylate monomers are not necessarily limited to direct radical addition.

To examine whether, in addition to homolytic bond cleavage, alternative and commonly encountered initiation pathways in acrylate photopolymerization could operate, further EPR experiments were performed. Mechanisms involving hydrogen atom transfer (HAT) or electron transfer (ET), which are frequently reported in photoinitiated radical processes, were considered. To probe these possibilities, experiments were designed based on the formation of semiquinone radicals, whose EPR signatures are well established and can be reliably observed [88,89,90,91,92,93].

Hydrogen atom transfer was investigated using 3,5-di-*tert*-butyl-*o*-hydroquinone (H_2_Q), a phenolic compound well known for its ability to donate hydrogen atoms in reactions with radical species [94,95,96]. In this case, abstraction of a hydrogen atom from H_2_Q leads to the formation of the corresponding 3,5-di-*tert*-butyl-*o*-semiquinone radical (SQ). Consequently, the observation of an SQ signal during the irradiation of photoinitiator 5 in the presence of H_2_Q would provide evidence supporting the hypothesis that HAT could be a potential initial step in the polymerization process.

The possibility of electron transfer (ET) from UV-generated radicals of photoinitiator 5 to the co-initiator/monomer as a potential initiation pathway was also investigated. For this purpose, 3,5-di-*tert*-butyl-*o*-benzoquinone (Q) was selected, as quinones are efficient electron acceptors that yield semiquinone radicals in this process [97,98,99,100]. It should be noted that one-electron reduction of Q yields exactly the same 3,5-di-*tert*-butyl-*o*-semiquinone radical as the one-electron oxidation of H_2_Q.

For these experiments, samples were prepared by dissolving 5 mg of H_2_Q or Q in 100 µL of 20 mM solution of photoinitiator 5. Control samples were prepared analogously, but with H_2_Q or Q dissolved in pure toluene. No EPR signals were detected in any of the control samples during 60 min of irradiation. Similarly, irradiation of the solution containing photoinitiator 5 and Q did not produce detectable spectra. In contrast, irradiation of the solution containing photoinitiator 5 and H_2_Q resulted in a weak but clear signal after approximately 15 min (Figure S2).

**Figure 5:**
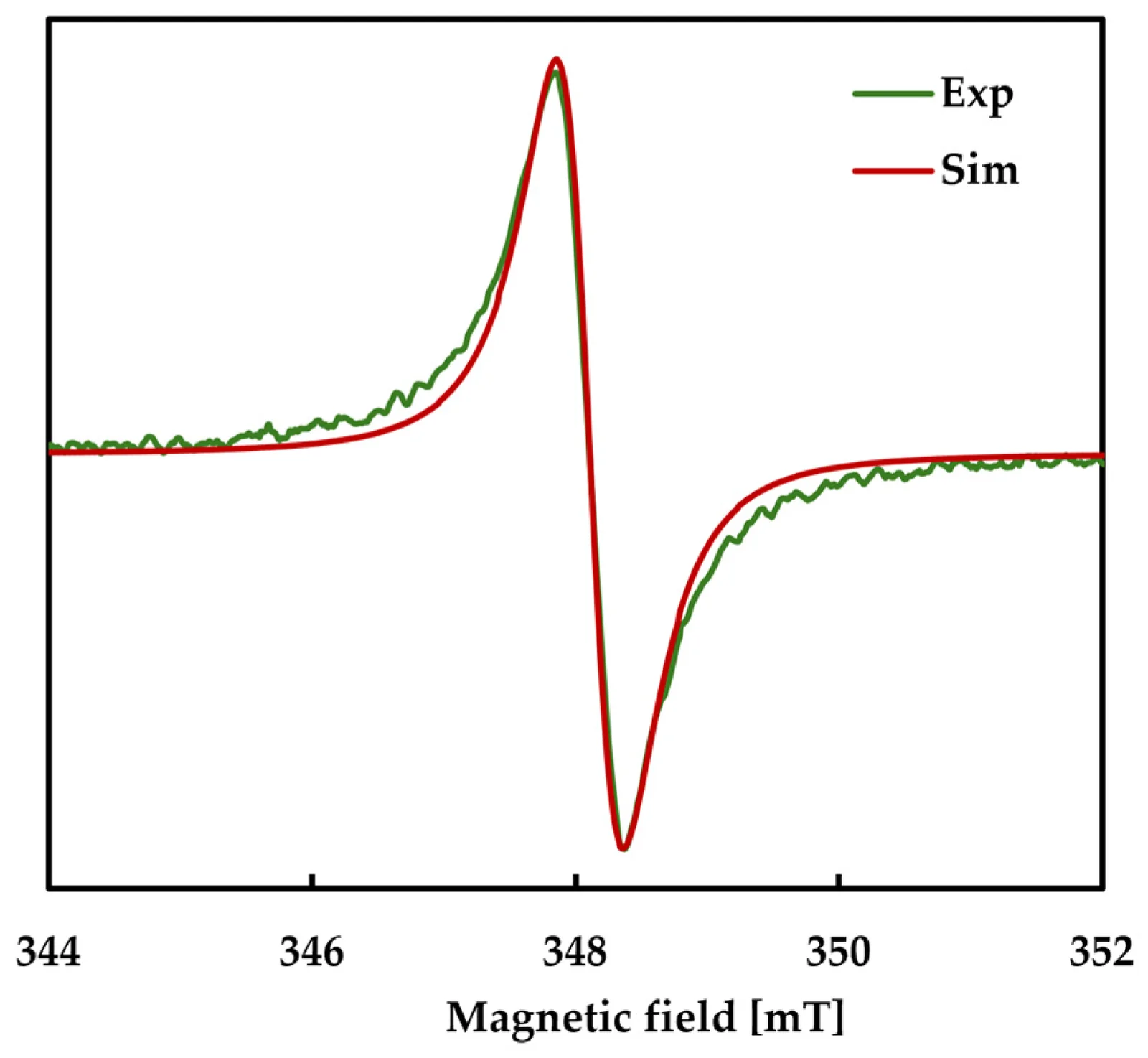
Experimental and simulated EPR spectra recorded as a 5-fold accumulation for the in situ UV-irradiated sample containing both H_2_Q and photoinitiator 5.

The spectrum shown in Figure 5 was unresolved due to hyperfine interactions, most likely caused by rapid spin relaxation and the large number of interacting nuclei (chemically nonequivalent hydrogen atoms in positions 4 and 6, as well as in two nonequivalent *tert*-butyl groups). However, the determined g_iso_ = 2.00462 is consistent with values reported for semiquinone radicals [69,89,90,91,92,93,100]. Taken together, these findings suggest that radicals generated by UV irradiation of photoinitiator 5 can initiate polymerization through hydrogen atom transfer rather than electron transfer: SQ was formed in the presence of H_2_Q, while no analogous signal was detected with Q as an electron acceptor.

However, although the radicals generated from photoinitiator 5 can abstract a hydrogen atom from reactive donors such as H_2_Q, the BDE values predicted for the tested monomers indicate that these compounds are not efficient hydrogen atom donors. Together with the results of experiments involving *N*-phenylglycine (NPG), this finding suggests that HAT is not the dominant pathway for activation of the investigated monomers. Nevertheless, the ability of the studied photoinitiators to initiate HAT processes, as demonstrated in experiments with H_2_Q, should be noted.

### 3.2. Photoinitiation Efficiency of Acrylate and Methacrylate Derivatives of 2-Mercaptobenzothiazole (1–10)

**Scheme 4:**
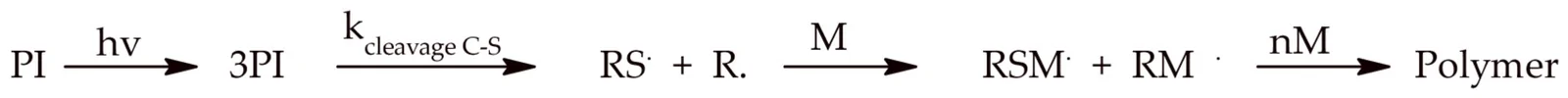
Initial stages of radical photopolymerization.

As demonstrated in the previous section, UV irradiation of the investigated acrylate and methacrylate derivatives of 2-mercaptobenzothiazole (1–10) leads to the formation of a thiyl radical (RS^•^) and a carbon-centered radical (R^•^). The generation of these reactive species provides a mechanistic basis for photoinitiation, as such radicals are generally known to participate in reactions with vinyl monomers. In acrylate polymerization, their interaction with the C=C bonds of the monomer (M) results in the formation of propagating radicals (RSM^•^, RM^•^) and, consequently, polymer growth (Scheme 4).

The photoinitiation efficiency of novel acrylate and methacrylate derivatives of 2-mercaptobenzothiazole (1–10) for activating the radical polymerization process of selected monomers (butyl acrylate (BA), 1,6-hexanediol diacrylate (HDDA), trimethylolpropane triacrylate (TMPTA), 2-hydroxyethyl acrylate (HEA) and pentaerythritol triacrylate (PETA) were studied.

#### 3.2.1. Butyl Acrylate (BA)

It was found that both acrylate (1–5) and methacrylate derivatives (6–10) of 2-mercaptobenzothiazole are incapable of initiating the polymerization of the monofunctional monomer butyl acrylate (BA). Irrespective of the photoinitiator concentration or the applied light intensities, only low heat flow release and significantly prolonged polymerization times were observed. The experimental findings suggest that the examined derivatives are incapable of efficiently generating propagating radicals for monofunctional butyl acrylate monomers under the experimental conditions applied.

#### 3.2.2. 2-Hydroxyethyl Acrylate (HEA)

In contrast to BA, 2-hydroxyethyl acrylate (HEA) had a significant effect on polymerization. The contrasting behaviors between butyl acrylate (BA) and 2-hydroxyethyl acrylate (HEA) arise from the chemical structures of both monomers. Although both monomers are monofunctional, they differ in that HEA contains a hydroxyl group. In the case of HEA, hydrogen bonds may form, which significantly affects the kinetics of polymerization, its rate, and chain mobility. The possibility of forming intermolecular hydrogen bonds causes the monomer molecules to become preliminarily organized, allowing the reactive acrylic bonds to be in close proximity to each other. This leads to an increase in the rate of propagation, unlike that of nonpolar butyl acrylate. At the same time, the increase in viscosity limits the mobility of the polymer chains, so the rate of the termination process drops dramatically, while the overall reaction rate increases.

It has been demonstrated that both acrylate (1–5) and methacrylate (6–10) derivatives of 2-mercaptobenzothiazole can initiate the photopolymerization of HEA. This assertion is corroborated by the results of calorimetric measurements. The kinetic parameters obtained, which are summarized in Table 4, demonstrate with high clarity that the investigated derivatives are effective photoinitiators in this system.

**Figure 6:**
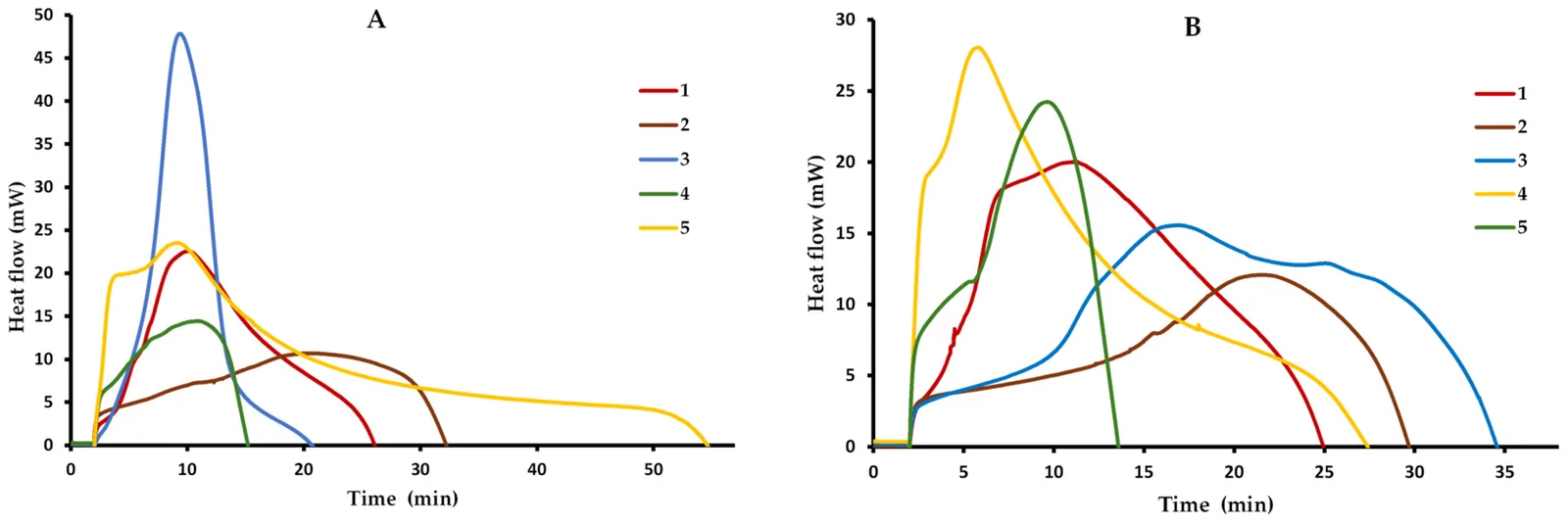
The dependence of heat release (mW) during HEA polymerization on irradiation time for acrylic derivatives of 2-mercaptobenzothiazole (1–5) at concentrations of 1% (**A**) and 5% (**B**) by weight.

As shown in Table 4, acrylate derivatives of 2-mercaptobenzothiazole (1–5) exhibited higher photoinitiation efficiency at lower concentrations, whereas the methacrylate derivatives (6–10) proved to be more effective at higher concentrations. According to Figure 6A, the highest heat flow release during HEA polymerization was observed in the presence of photoinitiator 3, while the lowest value was recorded for photoinitiator 2. It is noteworthy that a fivefold increase in the concentration of photoinitiator 3 resulted in a pronounced decrease in the total heat flow release and concomitant extension of the polymerization time (Figure 6B). An opposite trend was observed for polymerizing compositions containing chlorine-substituted photoinitiators (4 and 5). Higher heat flow release values were detected, and the polymerization proceeded at a faster rate.

**Figure 7:**
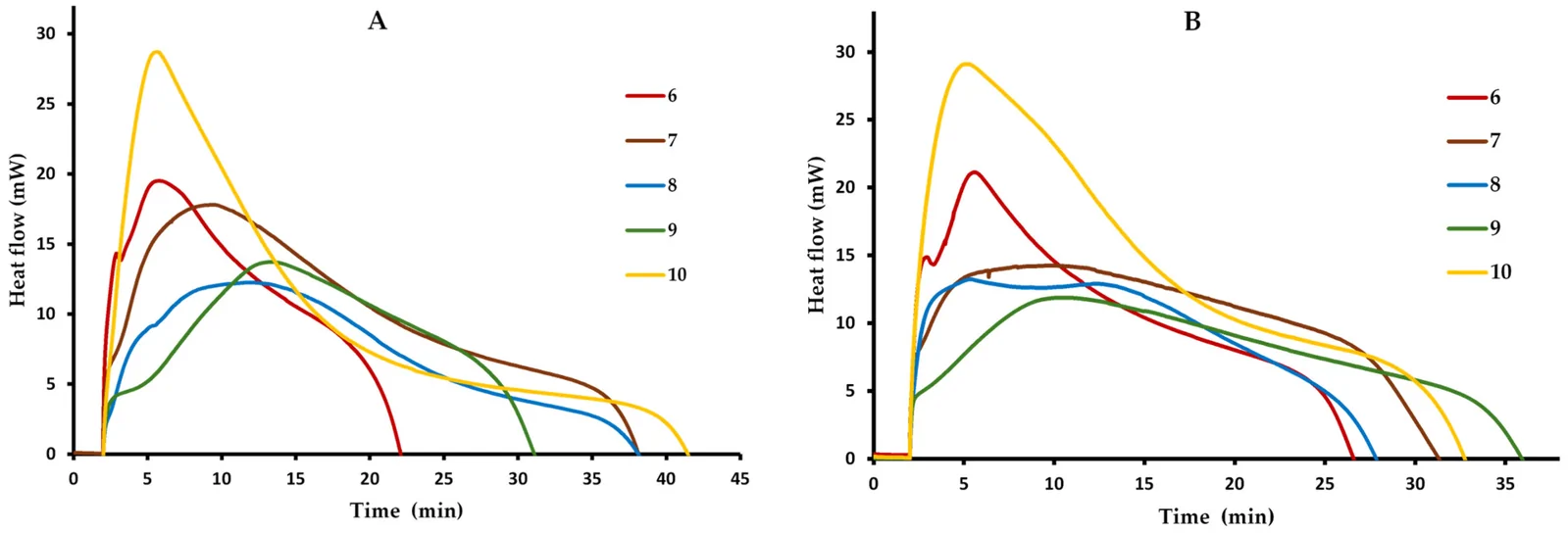
The dependence of heat release (mW) during HEA polymerization on irradiation time for methacrylic derivatives of 2-mercaptobenzothiazole (6–10) at concentrations of 5% (**A**) and 10% (**B**) by weight.

In the case of methacrylate derivatives of 2-mercaptobenzothiazole (6–10), it was observed that doubling the photoinitiator concentration in the polymerizing mixture (from 5% to 10%) did not significantly affect the amount of heat flow released during HEA polymerization; however, it shortened the polymerization time (Figure 7A,B).

**Figure 8:**
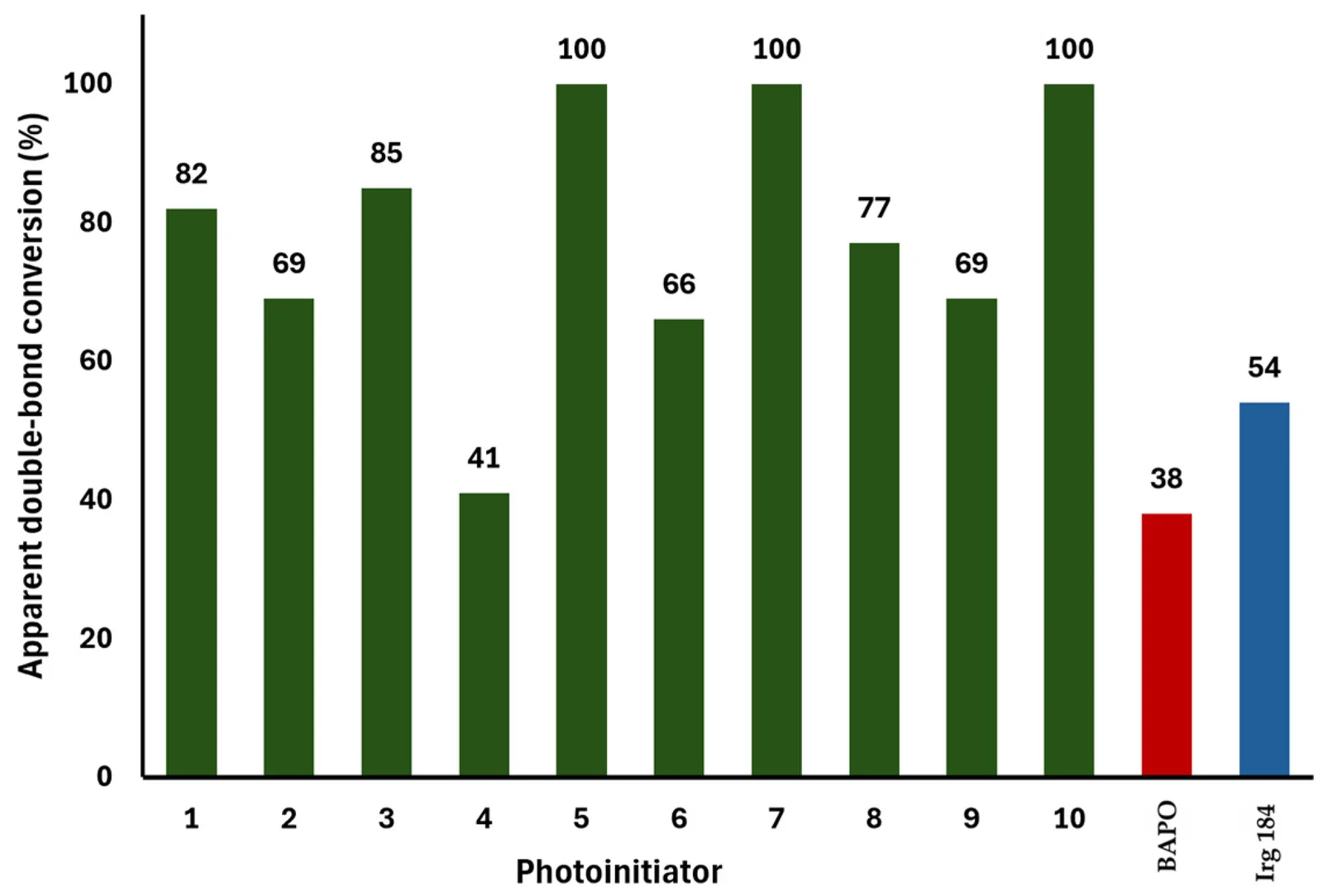
Apparent double-bond conversion obtained during the photopolymerization of 2-hydroxyethyl acrylate (HEA) initiated by acrylate and methacrylate derivatives of 2-mercaptobenzothiazole (1–10) and commercial photoinitiators BAPO and Irgacure 184 under identical irradiation conditions (1 wt.% photoinitiator concentration).

To assess the photoinitiating performance of the synthesized compounds under application-relevant conditions, additional photopolymerization experiments were carried out using two commercially available benchmark photoinitiators, Irgacure 819 (BAPO) and Irgacure 184, under identical irradiation conditions and at the same concentration (1 wt.%). The kinetic analysis revealed that the reference systems exhibit significantly higher rates of polymerization (R_p_), indicating more efficient generation of active centers at the early stage of the process (BAPO: R_p_ = 4.97 × 10^−2^ s^−1^; Irgacure 184: R_p_ = 3.26 × 10^−2^ s^−1^), compared to the studied photoinitiators (Table 4). This suggests that the commercial systems promote a more rapid onset of polymerization and faster initial chain growth. Despite this more favorable initial kinetic behavior, the final double-bond conversion values indicate a different overall efficiency of the process. The majority of the investigated 2-mercaptobenzothiazole derivatives afforded significantly higher monomer conversions in HEA than the commercial reference systems. While BAPO and Irgacure 184 reached final conversions of 38% and 54%, respectively, most of the synthesized derivatives yielded conversions ranging from 66% to 100%, demonstrating more complete utilization of the monomer (Figure 8).

#### 3.2.3. 1,6-Hexanediol Diacrylate (HDDA)

In the case of HDDA, the ability to initiate radical photopolymerization was found to be limited to derivatives substituted with an ethoxy group (1 and 6) and those containing a chlorine atom in the 6-position of the benzothiazole ring (5 and 10) (Table 5). Acrylate derivatives (1 and 5) demonstrated higher activity at lower concentrations (0.5–1%), as evidenced by relatively high Q_max_ values (43.8–64.4 mW) and initiation rates (I_p_) in the range of 1.28–1.80 × 10^−5^ s^−2^. In contrast, methacrylate derivatives (6 and 10) demonstrated enhanced efficiency at elevated concentrations (2–3%), with derivative 6 exhibiting the most favorable parameters: high Q_max_ (80.3–97.6 mW), rapid polymerization rates (R_p_ up to 4.71 × 10^−3^ s^−1^), and the highest initiation ability (I_p_ = 3.09 × 10^−5^ s^−2^).

For the indicated acrylic derivatives, the highest initiation ability was observed at low concentrations, suggesting efficient radical generation even under diluted conditions. In contrast, methacrylate derivatives, notably compound 10, exhibited remarkably low I_p_ values (0.51–0.64 × 10^−5^ s^−2^) across all concentrations examined, suggesting limited efficacy in the initiation phase despite substantial final apparent double-bond conversions (C_%_~25–31%). The marked superiority of compound 6 in comparison to 10 underscores the pivotal function of the electronic nature of substituents in regulating radical formation and stabilization.

#### 3.2.4. Trimethylolpropane Triacrylate (TMPTA)

In the case of the trifunctional monomer trimethylolpropane triacrylate (TMPTA), all acrylate derivatives of 2-mercaptobenzothiazole (1–5) could initiate the polymerization process (Table 6). The polymerization rates (R_p_) and the final apparent double-bond conversions (C_%_) indicate that these compounds exhibited satisfactory efficiency even at low concentrations (0.5–1%). Successive increases in initiator amount did not result in substantial improvements in the kinetic parameters, as confirmed by the example of derivative 2, where a tenfold increase in concentration (up to 10%) produced no meaningful enhancement in Q_max_, R_p_, or C_%_.

The analysis of the initiation ability I_p_ reveals distinct differences among the investigated acrylate derivatives. Photoinitiator 5 exhibited the highest initiation ability, with I_p_ values clearly exceeding those observed for the other derivatives, and also provided the highest apparent double-bond conversion at the highest investigated concentration. Compound 3 also showed high initiation ability, particularly at the lowest concentration, accompanied by high polymerization rates and apparent double-bond conversions. Compounds 1 and 2 exhibited moderate initiation ability, whereas compound 4 showed lower I_p_ values and generally lower apparent double-bond conversions. Overall, these results demonstrate that the photopolymerization performance of the investigated derivatives varies with their molecular structure, although the effect of photoinitiator concentration is not uniform across the series.

**Figure 9:**
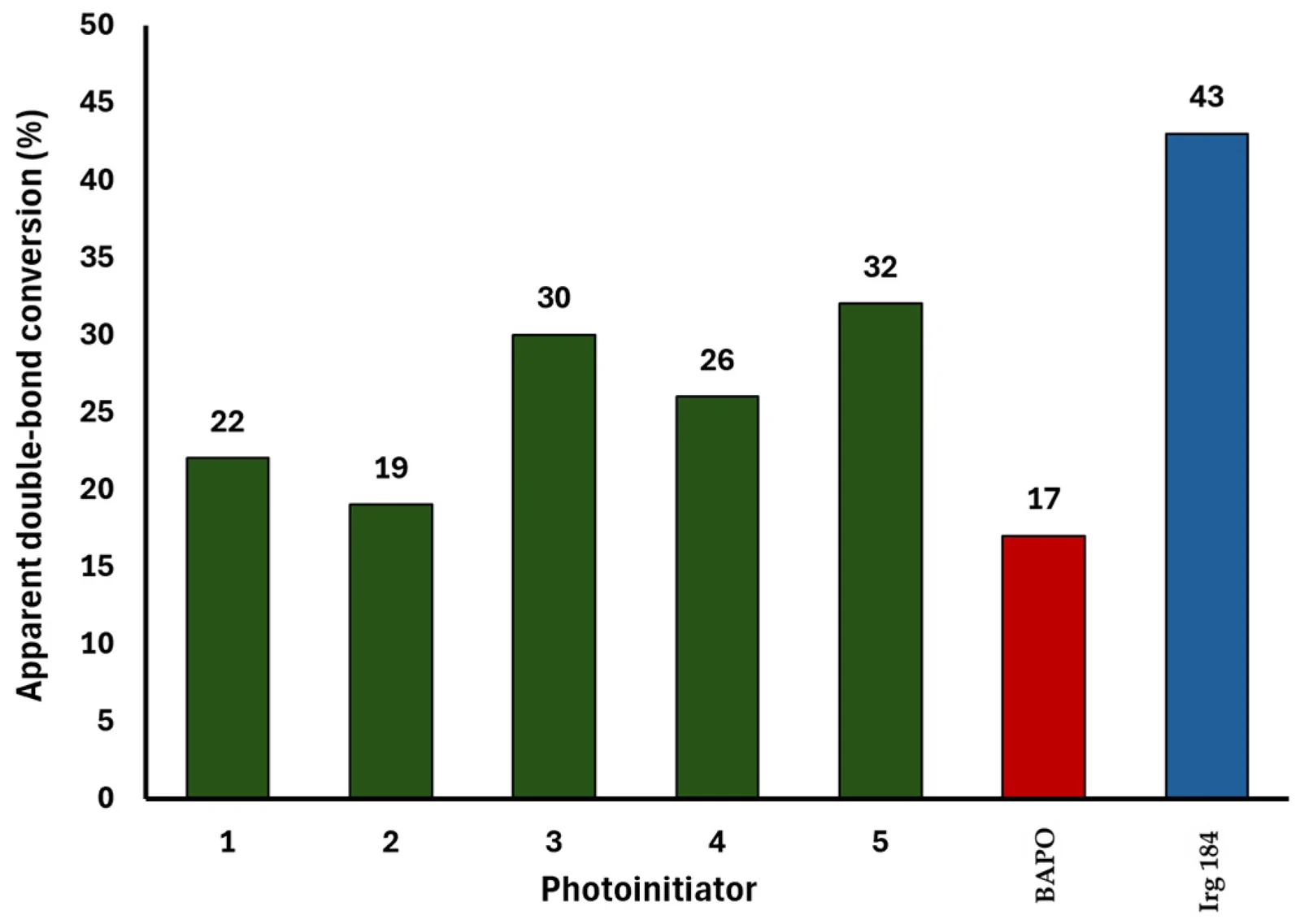
Comparison of the monomer conversion obtained in TMPTA photopolymerization initiated by synthesized acrylate derivatives of 2-mercaptobenzothiazole (1–5) and benchmark photoinitiators (BAPO and Irgacure 184) at 1 wt.% under identical irradiation conditions.

To evaluate the practical performance of the synthesized photoinitiators, additional photopolymerization experiments were carried out using two commercial benchmark photoinitiators, BAPO and Irgacure 184, under identical irradiation conditions and at the same concentration (1 wt.%). The comparison revealed distinct differences in the polymerization behavior of TMPTA. Both commercial photoinitiators exhibited substantially higher polymerization rates than the investigated 2-mercaptobenzothiazole derivatives. The R_p_ values determined for BAPO and Irgacure 184 were 37.17 × 10^−3^ and 25.02 × 10^−3^ s^−1^, respectively, whereas the synthesized derivatives afforded R_p_ values in the range of 0.06–1.60 × 10^−3^ s^−1^. However, the final monomer conversion did not follow the same trend. As shown in Figure 9, although BAPO generated radicals at the highest rate, it yielded the lowest final conversion (17%). In contrast, Irgacure 184 achieved the highest conversion (43%), while compounds 3 and 4 reached conversions of approximately 30% and 32%, respectively. A reasonable explanation for this trend is the rapid formation of a crosslinked network, which quickly shifts the reaction into a diffusion-controlled regime. Although the present study does not provide direct rheological evidence of this network immobilization, this phenomenon is frequently observed when highly efficient photoinitiators induce rapid, localized gelation, effectively trapping unreacted species.

**Figure 10:**
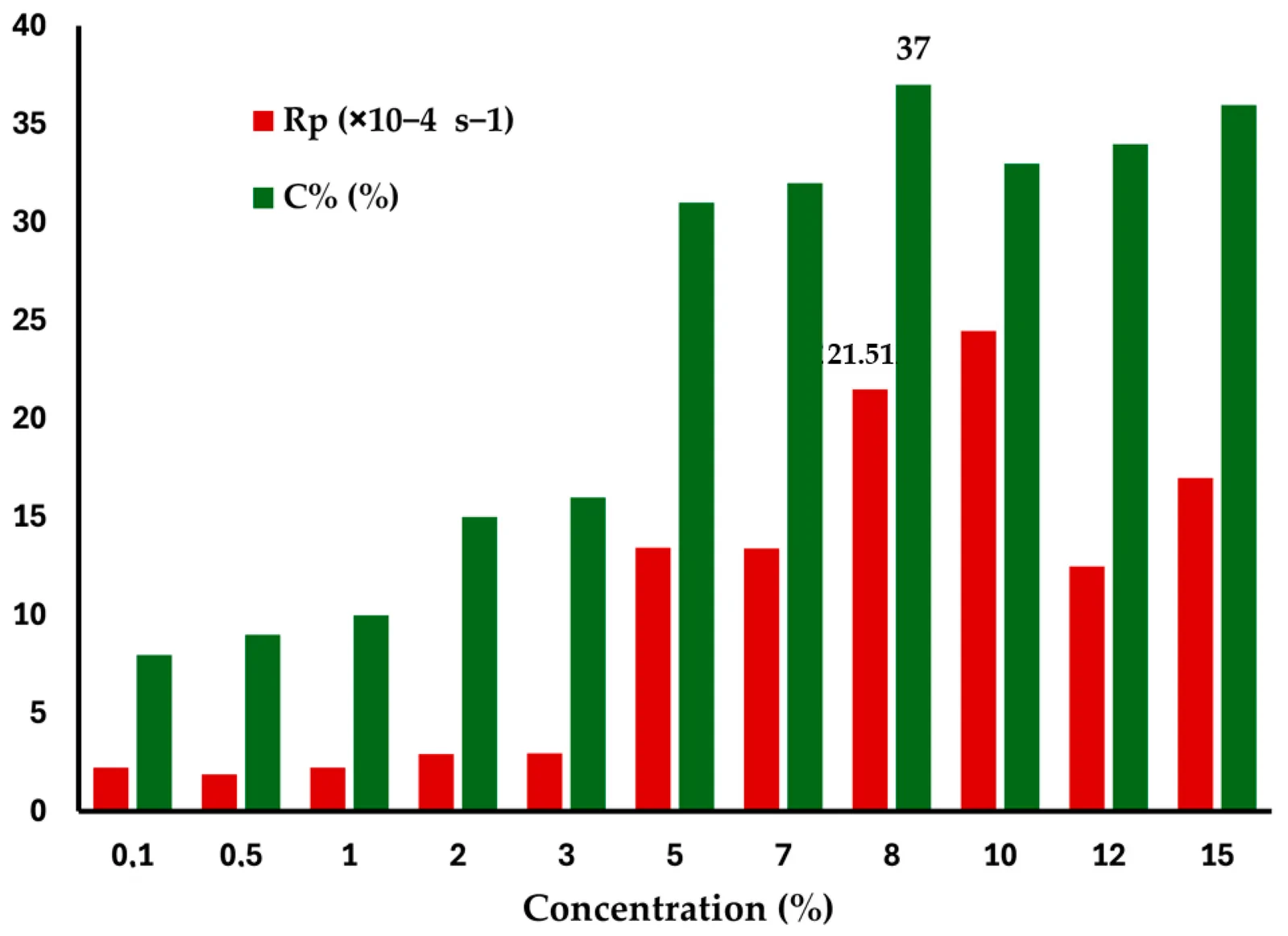
R_p_ and C_%_ values for methacrylic derivative 6 at differing weight concentrations in TMPTA.

For the methacrylate derivatives of 2-mercaptobenzothiazole (6–10), distinct differences in photoinitiation efficiency were observed. As demonstrated in Figure 10, the efficiency of TMPTA photopolymerization exhibited a strong dependence on the initiator concentration within the polymerizing mixture. It was observed that both the polymerization rate (R_p_) and the final conversion (C_%_) increased with initiator amount, reaching optimal values at an 8% concentration. At this point, R_p_ (2.15 × 10^−3^ s^−1^) and C_%_ (36.7%) were maximized. It was demonstrated that increases in concentration beyond a certain point did not result in improvements to the kinetic parameters.

According to the data in Table 7, it was found that derivatives 6 and 10 were the most efficient in initiating TMPTA polymerization, as indicated by their relatively high I_p_ values (14.63 and 10.79 × 10^−6^ s^−2^, respectively) and final apparent double-bond conversions exceeding 36%. In contrast, derivatives 7, 8, and 9 were markedly less effective, with efficiency decreasing in the order 7 > 8 > 9. These findings thus highlight that the least efficient initiators of TMPTA polymerization were derivatives containing a chlorine atom at the 5-position of the 2-mercaptobenzothiazole moiety, both among acrylate and methacrylate derivatives, whereas the most efficient were those substituted with either a chlorine atom or an ethoxy group at the 6-position.

#### 3.2.5. Pentaerythritol Triacrylate (PETA)

**Figure 11:**
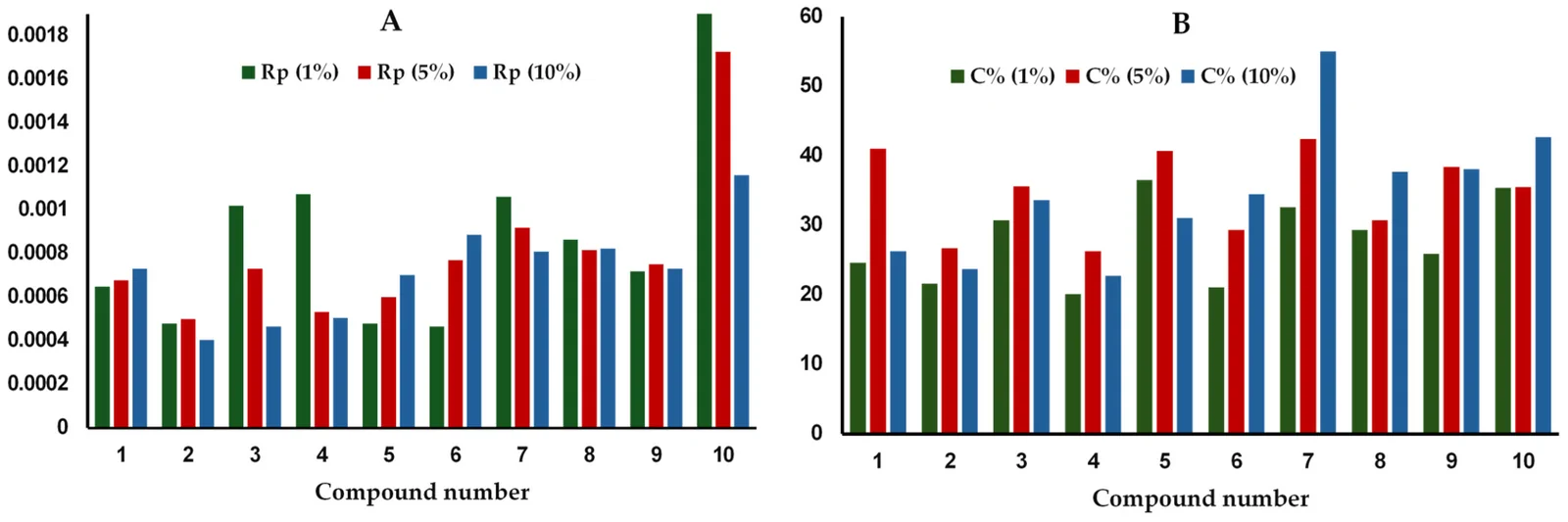
Rp (**A**) and C% (**B**) values for acrylic and methacrylic derivatives of 2-mercaptobenzothiazole at different weight concentrations (1%, 5% and 10%) in PETA.

In the case of the pentaerythritol triacrylate (PETA) used as a monomer, both the acrylate (1–5) and the methacrylate (6–10) derivatives of 2-mercaptobenzothiazole could initiate the process of photopolymerization. High polymerization rates (R_p_) were evident even at the lowest initiator concentration tested (1%) (Figure 11A).

Considering the final apparent double-bond conversions (C%), the systems with acrylate derivatives achieved the highest extent of double-bond conversion at an initiator concentration of 5%. In contrast, the methacrylate derivatives exhibited superior performance at 10% (Figure 11B). These findings suggest that acrylates and methacrylates require different optimal concentrations to be most effective in PETA formulations. These observations can be supported by analysis of the initiation ability parameter (I_p_) (Table 8). Acrylate derivatives demonstrated higher I_p_ values at lower concentrations, thereby confirming their capacity to efficiently generate initiating radicals under mild conditions. Conversely, methacrylate derivatives exhibited enhanced photoinitiation efficiency solely at higher concentrations. This behavior is most likely related to steric hindrance introduced by the additional methyl group adjacent to the double bond in methacrylates, which hampers the accessibility to the reactive C=C bond at lower initiator concentrations.

The results presented in Table 4, Table 5, Table 6, Table 7 and Table 8 reveal a distinct dependence of polymerization performance on photoinitiator concentration, with different trends observed for derivatives 1–5 and 6–10. Acrylate-containing photoinitiators 1–5 generally exhibit their highest initiation efficiencies at relatively low concentrations, whereas methacrylate-containing photoinitiators 6–10 tend to show higher polymerization rates and final conversions at increased concentrations. The opposite concentration trends observed for the two series may therefore be related to differences in the structural and photochemical properties of the acrylate- and methacrylate-containing photoinitiators.

Several factors may contribute to this concentration-dependent behavior. The incorporation of an acrylate or methacrylate moiety into the photoinitiator structure may affect its electronic properties, its excited-state behavior, and the reactivity of the species generated upon photolysis. Differences in the reactivity of the resulting radicals may consequently influence the balance between productive initiation, propagation, and competing termination or side reactions. Thus, an increase in the nominal photoinitiator concentration does not necessarily lead to a proportional increase in the concentration of radicals effectively contributing to polymerization.

At higher photoinitiator concentrations, inner filter effects may also become more pronounced. Increased absorption of the incident radiation by the photoinitiator can modify the spatial distribution of light within the polymerization mixture and, consequently, the distribution of radical generation throughout the sample. The magnitude of this effect may depend on the absorption properties and concentration of the individual compounds.

Furthermore, differences in the lifetimes of excited triplet states may affect the efficiency of the photochemical processes preceding S–C bond cleavage. Bimolecular quenching processes involving excited photoinitiator molecules or other components of the reaction mixture may also compete with productive bond dissociation, particularly at increased concentrations. The relative contribution of these processes may differ between the acrylate- and methacrylate-containing photoinitiators.

Accordingly, the opposite concentration dependencies observed for derivatives 1–5 and 6–10 may reflect differences in the relative contributions of competing photochemical and radical processes, together with the structural differences between the two photoinitiator series. Although the available data do not allow the contribution of each individual mechanism to be quantified, the results indicate that the optimum photoinitiator concentration is dependent on the structure of the photoinitiator and the polymerization system.

### 3.3. Structure–Activity Correlations for Photopolymerization Performance

The abundant experimental data on the photopolymerization performance of the studied 2-mercaptobenzothiazole derivatives prompted us to search for correlations between these data and molecular properties of compounds 1–10 (and the derived thiyl radicals) computed quantm-chemically. The following parameters, calculated at the M06-2X/def2-TZVP level of theory, were considered: TD-DFT vertical excitation energies to the S_1_ and T_1_ states, the S–C bond dissociation energies (BDEs) (taken as Gibbs free energies to correspond better to the experimental conditions), and Mulliken spin populations on the thiyl sulfur atom S_S_ and the thiazole nitrogen atom S_N_ (and their linear combinations S_S_+S_N_ and S_S_-S_N_). The spin density was first analyzed for each compound and the two atoms stated above were observed to undergo the most significant changes in the series of derivatives. Interestingly, the thiazole sulfur atom had a small and barely changing Mulliken spin population. The electronic substituent effects were also included in the study, in the form of the classical Hammett constants [101]. It must be noted at this point that Hammett constants were derived for ionic processes and acid/base equilibria, and are not directly related to the radical spin populations. However, the phenyl ring preserved in the structures can be treated as a transmitter of the substituent effect to other parts of the molecule, allowing indirect electron density redistribution. On the basis of the observed spin density changes and location of the R_1_/R_2_ substituents (see Scheme 1), the σ_p_ constants were considered, with the exception of compounds 4 and 9, for which the σ_m_ parameters were taken. The set of computational descriptors is provided in the Table S4 of the Supplementary Materials. Extensive correlation tables are also provided in the Supplementary Materials in Tables S5–S9.

Correlation analysis for the photopolymerization of HEA, summarized in Table S5, shows that two computational descriptors correlate relatively well with the experimental data: the S_1_ excitation energy (reaching correlation coefficient R = −0.5951) and the polymerization rate R_p_ (with values close to −0.5 for the apparent double-bond conversion, C%, at different reaction times). The Hammett constant correlates less well with the experimental data.

The case of TMPTA monomers was divided into three sub-cases: photoinititation by acrylate series 1–5 (Table S6) and methacrylate series 6–10 (Table S7), and the overall combined performance of the studied derivatives 1–10 (Table S8). This comparison highlights the fact that correlations within a coherent series are usually stronger than for a combined set. In both acrylate and methylacrylate cases the role of the S_1_ (but not T_1_) excitation energy descriptor is important (correlations reaching R = 0.71 for the maximum heat release time t_max_), and correlations of similar strength are found for the S–C bond dissociation energy and the spin density parameters. Negative correlation coefficients of the kinetic parameters with the BDE reflect the fact that the weaker S–C bond promotes a faster process. The Hammett substituent constants are less important for the 1–5 series (R values not exceeding 0.21) than for methylacrylate derivatives 6–10. Interestingly, when the combined set of compounds is considered (Table S8), the correlation coefficients become significantly closer to 0, but the prominent role of the BDE value is retained.

The data for the photoinduced polymerization of PETA do not exhibit strong correlations between the experimental and calculated data (see Table S9).

The results of the search for structure–photopolymerization efficiency correlations can be summarized with the following observations:

- Quantitative correlations, measured using the classical correlation coefficient, are of diverse strength. The best correlations are found for the photoinduced polymerization of TMPTA, some weaker relations are visible for HEA, and the case of PETA shows the worst correlation between the experimental values and calculations.
- The most important molecular descriptors include S_1_ vertical excitation energy, the Mulliken spin population on the thiyl sulfur atom or the thiazole nitrogen atom, and in case of TMPTA, the Hammett substituent constants.
- The acrylate and methacrylate series of compounds (1–5 and 6–10 respectively) exhibit stronger structure–activity correlations when studied separately, not together.
- The experimental properties which are best correlated with the computational data, for the best case (TMPTA), are the polymerization rates (Rp) and initiation ability parameters (Ip).

## 4. Conclusions

The photochemically initiated radical polymerization of selected acrylate monomers was investigated using a series of ten newly synthesized acrylate and methacrylate derivatives of 2-mercaptobenzothiazole as copolymerizable photoinitiators. Quantum-chemical calculations revealed that, for all investigated compounds, homolytic cleavage of the S–C bond constitutes the preferred photochemical pathway, leading to the formation of thiyl (RS^•^) and carbon-centered radicals (R^•^). These theoretical predictions were fully supported by EPR measurements performed under in situ UV irradiation conditions, confirming the generation of the expected radical species.

An evaluation of photoinitiation efficiency based on polymerization kinetics determined by differential scanning calorimetry demonstrated that the performance of the investigated photoinitiators strongly depends not only on the acrylate or methacrylate character of the derivative, but also on the nature and position of substituents within the 2-mercaptobenzothiazole moiety. It was observed that not all compounds exhibited comparable efficiency, indicating that subtle structural variations play a decisive role in determining initiation performance. In particular, derivatives bearing a chlorine substituent at the 5-position of the benzothiazole ring consistently showed the lowest photoinitiation efficiency across all investigated systems. In contrast, derivatives substituted with an ethoxy group or chlorine at the 6-position generally exhibited enhanced performance in most monomer–photoinitiator combinations.

Among the studied monomers, the highest polymerization efficiency was observed for formulations containing 2-hydroxyethyl acrylate (HEA), as well as trifunctional acrylates, namely trimethylolpropane triacrylate (TMPTA) and pentaerythritol triacrylate (PETA). Additional benchmark experiments performed using the commercial photoinitiators BAPO and Irgacure 184 further demonstrated that the photoinitiating performance of the investigated derivatives strongly depends on the monomer structure. In particular, several synthesized photoinitiators afforded higher final monomer conversions than the commercial reference systems in HEA photopolymerization, whereas compounds 3 and 4 exhibited competitive performance in TMPTA. These findings highlight the importance of monomer structure and functionality in determining the overall efficiency of the photopolymerization process and suggest favorable interactions between the generated radical species and these monomers.

The experimental and theoretical approaches employed in this study, when considered as a whole, provide a comprehensive understanding of the structure–reactivity relationships governing the performance of acrylate and methacrylate derivatives of 2-mercaptobenzothiazole as photoinitiators. While correlations between experimental photopolymerization parameters and computational descriptors were semi-quantitative, they allowed for determining the most important features to be investigated in rational design of new photoinitiators of the benzothiazole family. Depending on the monomer, these parameters are S_1_ excitation energy, S–C bond dissociation energy or spin populations in the thiyl and thiazole moieties. The results of this study identify a new promising and modifiable class of Type I polymerization unsaturable 2-mercaptobenzothiazole-based photoinitiators for radical photopolymerization of acrylate monomers.

## Figures and Tables

**Table 1:** Relative energies (kcal/mol) of radicals formed via different fragmentation paths calculated at the M06-2X/def2-TZVP and CAM-B3LYP/def2-TZVP levels of theory. Path 2 was consistently the preferred (lowest energy) pathway.

No.	ΔE (kcal/mol) (M06-2X/def2-TZVP)					ΔE (kcal/mol) (CAM-B3LYP/def2-TZVP)				
	Path 1	Path 2	Path 3	Path 4	Path 5	Path 1	Path 2	Path 3	Path 4	Path 5
1	16.57	0	16.62	28.64	39.84	17.92	0	17.44	27.58	43.68
2	15.21	0	15.19	22.45	38.36	16.62	0	16.08	26.08	42.25
3	14.30	0	21.59	26.05	37.38	15.75	0	15.18	25.06	41.30
4	14.63	0	14.36	26.05	37.55	15.46	0	14.61	24.30	40.73
5	14.04	0	13.74	25.34	36.85	16.05	0	15.23	24.99	41.40
6	17.02	0	17.33	28.70	37.19	18.41	0	18.04	26.66	39.41
7	15.04	0	15.35	26.53	35.04	16.57	0	16.19	24.59	37.38
8	14.14	0	14.42	25.47	34.06	15.71	0	15.30	23.57	36.43
9	14.46	0	14.51	25.48	34.23	15.41	0	14.72	22.81	35.86
10	13.87	0	13.88	24.76	33.53	16.00	0	15.35	23.50	36.53

**Table 2:** Calculated vertical excitation energies and S–C electronic bond dissociation energies for acrylate and methacrylate derivatives of 2-mercaptobenzothiazole (1–10).

No.	S_1_ Energy (kcal/mol)		T_1_ Energy (kcal/mol)		BDE ^a^ (kcal/mol)	
	M06-2X ^b^	CAM-B3LYP ^c^	M06-2X ^b^	CAM-B3LYP ^c^	M06-2X ^b^	CAM-B3LYP ^c^
1	108.76	107.84	80.96	70.67	70.47	62.16
2	112.61	112.03	73.02	72.92	71.99	63.64
3	113.47	112.92	85.18	73.66	73.01	64.64
4	110.03	109.74	84.43	72.75	72.97	65.34
5	110.60	110.16	82.70	71.54	73.67	64.65
6	109.66	108.84	82.58	72.29	70.10	61.69
7	112.58	111.99	73.02	72.92	72.27	63.74
8	113.44	112.88	85.18	78.80	73.29	64.73
9	110.00	109.71	84.43	72.75	73.23	65.42
10	110.57	110.12	82.70	71.54	73.94	64.74

^a^ The S–C bond dissociation energies. ^b^ TD-DFT calculations were performed at the M062X/def2-TZVP level (5 excited states, single-point). ^c^ TD-DFT calculations were performed at the CAM-B3LYP/def2-TZVP level (5 excited states, single-point).

**Table 3:** The values of electronic BDEs for the weakest C–H bonds in the monomers calculated at the M06-2X/def2-TZVP and CAM-B3LYP/def2-TZVP levels of theory.

Monomer	BDE (kcal/mol)	
	M06-2X/def2-TZVP	CAM-B3LYP/def2-TZVP
BA	103.39	101.87
HDDA	103.40	101.87
TMPTA	102.43	100.93
HEA	102.37	100.72
PETA	99.75	98.87

**Table 4:** Kinetic parameters of radical polymerization of HEA initiated by acrylate and methacrylate derivatives of 2-mercaptobenzothiazole (1–10).

No.	Concentration ^a^ (%)	R_p_ (10^−4^ s^−1^)	C_%_ (%)	C_%_ (%) After 10 min	C_%_ (%) After 15 min	C_%_ (%) After 20 min
1	1 5 10	11.16 9.91 8.58	82.4 84.4 79.1	28.1 29.1 23.0	56.2 56.9 47.4	72.7 71.8 64.9
2	1 5 10	5.29 5.97 2.78	68.8 58.9 42.2	12.1 9.6 6.0	23.6 18.5 12.5	38.2 32.7 22.4
3	1 5 10	23.67 7.70 4.25	85.3 93.8 45.0	66.9 10.4 9.7	80.7 28.6 16.4	85.2 49.1 24.7
4	1 5 10	7.14 11.99 13.86	41.2 52.5 57.9	24.4 36.1 36.3	41.2 ^c^ 52.5 ^d^ 57.9 ^e^	- - -
5	1 5 10	11.64 13.88 12.66	^b^ 93.9 83.5	46.1 51.7 49.1	74.3 71.7 66.9	93.5 84.2 78.4
6	1 5 10	6.86 9.66 10.45	66.2 73.7 81.7	26.3 39.4 40.0	43.9 58.2 57.9	59.9 71.4 71.2
7	1 5 10	10.56 8.81 7.05	^b^ ^b^ 94.5	35.3 33.4 30.0	62.5 57.4 50.4	81.4 75.6 68.4
8	1 5 10	3.90 6.07 6.55	77.3 83.1 75.4	6.5 21.6 28.6	15.6 40.4 47.4	26.9 55.7 62.7
9	1 5 10	5.65 6.79 5.88	69.4 78.3 81.8	9.7 16.2 20.5	19.5 35.8 37.6	33.9 53.9 52.6
10	1 5 10	15.25 14.21 14.03	^b^ ^b^ ^b^	49.3 52.5 56.2	72.5 76.2 82.3	86.0 90.4 98.2

^a^ The weight concentration related to 1 g of polymerizing mixture. ^b^ The complete conversion of the monomer. ^c^ The total polymerization time was 15.19 min. ^d^ The total polymerization time was 13.65 min. ^e^ The total polymerization time was 14.45 min.

**Table 5:** Kinetic parameters of radical polymerization of HDDA initiated by acrylate (1, 5) and methacrylate (6, 10) derivatives of 2-mercaptobenzothiazole.

No	Concentration ^a^ (%)	Q_max_ (mW)	t_max_ (min)	R_p_ (10^−3^ s^−1^)	I_p_ (10^−5^ s^−2^)	C_%_ (%)
1	0.5 1 2 3	43.79 34.90 51.55 43.63	2.75 3.30 2.72 2.63	2.11 1.68 2.49 2.10	1.28 0.85 1.52 1.33	24.3 22.8 22.6 21.9
5	0.5 1 2 3	64.38 50.00 37.90 35.72	2.87 2.60 2.69 2.60	3.10 2.41 1.83 1.72	1.80 1.55 1.13 1.11	35.3 32.1 25.0 23.2
6	0.5 1 2 3	68.65 58.57 80.33 97.61	2.41 2.56 2.60 2.53	3.31 2.82 3.87 4.71	2.29 1.84 2.49 3.09	24.6 26.3 32.4 36.7
10	0.5 1 2 3	29.56 27.01 25.48 21.69	3.69 4.00 3.83 3.43	1.43 1.30 1.23 1.05	0.64 0.54 0.53 0.51	27.3 28.9 30.8 24.5

^a^ The weight concentration related to 1 g of polymerizing mixture.

**Table 6:** Kinetic parameters of radical polymerization of TMPTA initiated by acrylate derivatives of 2-mercaptobenzothiazole (1–5).

No	Concentration ^a^ (%)	Q_max_ (mW)	t_max_ (min)	R_p_ (10^−3^ s^−1^)	I_p_ (10^−6^ s^−2^)	C_%_ (%)
1	0.5 1 2 3	18.31 21.64 24.38 25.57	5.79 7.11 5.75 4.73	0.77 0.91 1.03 1.08	2.22 3.06 2.97 3.79	24.7 21.8 26.5 29.7
2	0.5 1 2 3 10	13.64 12.97 14.03 13.53 12.76	5.47 7.26 6.06 6.83 6.23	0.57 0.55 0.59 0.57 0.54	1.75 1.23 1.63 1.39 1.44	19.8 18.7 19.4 22.0 21.3
3	0.5 1 2 3	33.13 27.26 24.48 20.52	3.99 4.81 5.70 6.81	1.39 1.15 1.03 0.86	5.82 3.97 3.01 2.12	30.5 30.3 34.3 36.8
4	0.5 1 2 3	13.49 14.69 10.67 13.11	8.35 8.52 9.94 6.15	0.57 0.61 0.45 0.55	1.13 1.19 0.75 1.50	24.4 26.0 16.3 21.7
5	0.5 1 2 3	28.26 36.76 35.82 38.63	4.67 3.62 3.73 3.42	1.19 1.55 1.51 1.63	4.25 7.13 6.74 7.93	33.2 32.3 32.3 39.3

^a^ The weight concentration related to 1 g of polymerizing mixture.

**Table 7:** Kinetic parameters of radical polymerization of TMPTA initiated by methacrylate derivatives of 2-mercaptobenzothiazole (6–10).

No	Concentration ^a^ (%)	Q_max_ (mW)	t_max_ (min)	R_p_ (10^−3^ s^−1^)	I_p_ (10^−6^ s^−2^)	C_%_ (%)
6	8	51.09	2.45	2.15	14.63	36.7
7	8	19.53	4.31	0,82	3.18	34.4
8	8	25.83	4.81	1.09	3.77	32.2
9	8	15.51	4.43	0.65	2.46	25.0
10	8	47.01	3.06	1.98	10.79	37.5

^a^ The weight concentration related to 1 g of polymerizing mixture.

**Table 8:** Kinetic parameters of radical polymerization of PETA initiated by acrylate and methacrylate derivatives of 2-mercaptobenzothiazole (1–10).

No	Concentration ^a^ (%)	Q_max_ (mW)	t_max_ (min)	R_p_ (10^−4^ s^−1^)	I_p_ (10^−6^ s^−2^)	C_%_ (%)
1	1 5 10	15.33 16.00 17.24	9.25 8.06 6.49	6.50 6.78 7.31	1.17 1.40 1.88	24.6 41.0 26.3
2	1 5 10	11.32 11.79 9.52	9.85 11.21 10.12	4.80 5.00 4.04	0.81 0.74 0.66	21.6 26.7 23.7
3	1 5 10	24.1 17.27 11.01	4.74 7.27 10.48	10.21 7.32 4.66	3.59 1.68 0.74	30.7 35.6 33.6
4	1 5 10	11.37 12.55 11.91	9.05 8.12 8.39	4.82 5.32 5.05	0.89 1.09 1.00	20.1 26.3 22.7
5	1 5 10	25.31 14.83 16.57	4.70 7.04 4.08	10.73 6.01 7.02	3.80 1.42 2.87	36.5 40.7 31.0
6	1 5 10	11.01 18.21 20.94	7.85 5.43 6.19	4.67 7.71 8.88	0.99 2.37 2.39	21.1 29.3 34.5
7	1 5 10	25.04 21.67 19.09	4.24 3.61 3.60	10.61 9.19 8.09	4.17 4.24 3.75	32.6 42.4 55.0
8	1 5 10	20.41 19.26 19.41	6.34 5.98 6.27	8.65 8.16 8.23	2.27 2.27 2.19	29.3 30.7 37.7
9	1 5 10	16.97 17.74 17.25	6.81 5.37 5.37	7.19 7.52 7.31	1.76 2.33 2.27	25.9 38.4 38.1
10	1 5 10	34.97 40.72 27.37	6.27 3.35 3.79	14.82 17.26 11.60	3.94 8.58 5.11	35.4 45.8 42.7

^a^ The weight concentration related to 1 g of polymerizing mixture.

## Data Availability

The original contributions presented in this study are included in the article and Supplementary Materials. Further inquiries can be directed to the corresponding author.

## Supplementary Materials

The following supporting information can be downloaded at: https://www.mdpi.com/article/10.3390/polym18212613/s1, Table S1. Relative energies (kcal/mol) of radicals formed via different fragmentation paths calculated at the M06-2X/def2-TZVP and CAM-B3LYP/def2-TZVP levels of theory. Path 2 was consistently the preferred (lowest energy) pathway (black); electronic + thermal (ZPE, rotational, translational) energies (blue) and electronic + thermal Gibbs free enthalpies (red); Table S2. Calculated vertical excitation energies and S–C bond dissociation energies for acrylate and methacrylate derivatives of 2-mercaptobenzothiazole (1–10). The S–C BDE parameters are derived from: electronic energies (black), electronic + thermal (ZPE, rotational, translational) energies (blue) and electronic + thermal Gibbs free enthalpies (red); Table S3. The values of BDEs for the weakest C–H bonds in the monomers calculated at the M06-2X/def2-TZVP and CAM-B3LYP/def2-TZVP levels of theory (black); electronic + thermal (ZPE, rotational, translational) energies (blue) and electronic + thermal Gibbs free enthalpies (red); Figure S1. Experimental EPR spectra (common intensity scale) recorded for the in situ UV-irradiated sample containing both PBN and photoinitiator 5; Figure S2. Experimental EPR spectra (common intensity scale) recorded for the in situ UV-irradiated sample containing both H_2_Q and photoinitiator 5; Table S4. Computational descriptors used within the correlation study: S_0_→S_1_ and S_0_→T_1_ vertical excitation energies; S–C bond dissociation energies (BDEs, given as Gibbs free enthalpies); Mulliken spin populations at the thiyl S (S_S_) and thiazole N (S_N_) of the thiyl radicals; and the spin population sum and difference, together with classical Hammett constants σ of benzothiazole substituents, for acrylate (1–5) and methacrylate (6–10) derivatives of 2-mercaptobenzothiazole; Table S5. Correlation coefficients between the computational descriptors and the experimental data from Table 4 of the main manuscript (kinetic parameters of radical polymerization of HEA initiated by compounds 1–10; data for C=10% initiator concentration were chosen); Table S6. Correlation coefficients between the computational descriptors and the experimental data from Table 6 of the main manuscript (kinetic parameters of radical polymerization of TMPTA initiated by compounds 1–5; data for C=3% initiator concentration were chosen); Table S7. Correlation coefficients between the computational descriptors and the experimental data from Table 7 of the main manuscript (kinetic parameters of radical polymerization of TMPTA initiated by compounds 6–10; data for C=8% initiator concentration were chosen); Table S8. Correlation coefficients between the computational descriptors and the combined experimental data from Table 6 and Table 7 of the main manuscript (kinetic parameters of radical polymerization of TMPTA initiated by compounds 1–10; data for initiator concentration C=3% for compounds 1–5 and 8% for compounds 6–10 were chosen); Table S9. Correlation coefficients between the computational descriptors and the experimental data from Table 8 of the main manuscript (kinetic parameters of radical polymerization of PETA initiated by compounds 1–10; data for C=10% initiator concentration were chosen).

## Abbreviations

The following abbreviations are used in this manuscript:

ITX	Isopropylthioxanthone
CQ	Camphorquinone
BPO	Benzoyl peroxide
MBT	2-mercaptobenzothiazole
BTDS	2,2′-dithiobisbenzothiazole
EPR	Electron Paramagnetic Resonance
DSC	Differential Scanning Calorimetry
TD-DFT	Time-Dependent Density Functional Theory
DFT	Density Functional Theory
BDE	Bond Dissociation Energy
PES	Potential Energy Surface
PBN	*N*-*tert*-butyl-*α*-phenylnitrone
H_2_Q	3,5-di-*tert*-butyl-*o*-hydroquinone
Q	3,5-di-*tert*-butyl-*o*-benzoquinone
BA	Butyl Acrylate
HDDA	1,6-hexanediol diacrylate
TMPTA	Trimethylolpropane triacrylate
HEA	Hydroxyethyl acrylate
PETA	Pentaerythritol triacrylate
ISC	Intersystem Crossing
NPG	*N*-phenylglycine
HAT	Hydrogen Atom Transfer
ET	Electron Transfer
