# Supplementary material for: Bridging Experiment and Theory: Insights into the Photoinitiation Efficiency of Acrylate and Methacrylate Derivatives of 2-Mercaptobenzothiazole in Radical Photopolymerization of Selected Acrylates
Source: Polymers (Basel). 2026 Oct 22;18(21):2613. doi: 10.3390/polym18212613 (PMC13765695; doi:10.3390/polym18212613)
Supplement: Supplementary file 1 [file polymers-18-02613-s001.zip › polymers-4563217-supplementary.pdf]

Supplementary Materials

Bridging Experiment and Theory: Insights into the Photoinitiation Efficiency of Acrylate and Methacrylate Derivatives of 2-Mercaptobenzothiazole in Radical Photopolymerization of Selected Acrylates.

Agnieszka Skotnicka [1*], Maciej Witwicki [2], Jarosław J. Panek [2], Aneta Jezierska [2], Janina Kabatc-Borcz [1], Adrian A. Chelewski.

[1] Faculty of Chemical Technology and Engineering, Bydgoszcz University of Science and Technology, Seminaryjna 3, 85-326 Bydgoszcz, Poland

[2] University of Wrocław, Faculty of Chemistry, ul. F. Joliot-Curie 14, 50-383 Wrocław, Poland

***Correspondence should be addressed to:** Agnieszka Skotnicka; e-mail: Agnieszka.Skotnicka@pbs.edu.pl

Table of contents:

1. **Table S1.** Relative energies (kcal/mol) of radicals formed via different fragmentation paths calculated at the M06-2X/def2-TZVP and CAM-B3LYP/def2-TZVP levels of theory. Path 2 was consistently the preferred (lowest energy) pathway (black) electronic + thermal (ZPE, rotational, translational) energies (blue) and electronic + thermal Gibbs free enthalpies (red).
2. **Table S2.** Calculated vertical excitation energies and S-C bond dissociation energies for acrylate and methacrylate derivatives of 2-mercaptobenzothiazole (1-10). The S–C BDE parameters are derived from: electronic energies (black), electronic + thermal (ZPE, rotational, translational) energies (blue) and electronic + thermal Gibbs free enthalpies (red).
3. **Table S3.** The values of BDEs for the weakest C–H bonds in the monomers calculated at the M06-2X/def2-TZVP and CAM-B3LYP/def2-TZVP levels of theory (black) electronic + thermal (ZPE, rotational, translational) energies (blue) and electronic + thermal Gibbs free enthalpies (red).
4. **Figure S1.** Experimental EPR spectra (common intensity scale) recorded for the *in situ* UV-irradiated sample containing both PBN and photoinitiator 5.

5. **Figure S2.** Experimental EPR spectra (common intensity scale) recorded for the *in situ* UV-irradiated sample containing both H_2Q and photoinitiator 5.
6. **Table S4.** Computational descriptors used within the correlation study: $S_0 \rightarrow S_1$ and $S_0 \rightarrow T_1$ vertical excitation energies, S–C bond dissociation energies (BDE, given as Gibbs free enthalpies), Mulliken spin populations at the thiyl S (S_S) and thiazole N (S_N) of the thiyl radicals, the spin population sum and difference, together with classical Hammett constants σ of benzothiazole substituents, for acrylate (1-5) and methacrylate (6-10) derivatives of 2-mercaptobenzothiazole.
7. **Table S5.** Correlation coefficients between the computational descriptors and the experimental data from Table 4 of the main manuscript (kinetic parameters of radical polymerization of HEA initiated by compounds 1-10; data for C=10% initiator concentration were chosen).
8. **Table S6.** Correlation coefficients between the computational descriptors and the experimental data from Table 6 of the main manuscript (kinetic parameters of radical polymerization of TMPTA initiated by compounds 1-5; data for C=3% initiator concentration were chosen).
9. **Table S7.** Correlation coefficients between the computational descriptors and the experimental data from Table 7 of the main manuscript (kinetic parameters of radical polymerization of TMPTA initiated by compounds 6-10; data for C=8% initiator concentration were chosen).
10. **Table S8.** Correlation coefficients between the computational descriptors and the combined experimental data from Tables 6 and 7 of the main manuscript (kinetic parameters of radical polymerization of TMPTA initiated by compounds 1-10; data for initiator concentration C=3% for compounds 1-5 and 8% for compounds 6-10 were chosen).
11. **Table S9.** Correlation coefficients between the computational descriptors and the experimental data from Table 8 of the main manuscript (kinetic parameters of radical polymerization of PETA initiated by compounds 1-10; data for C=10% initiator concentration were chosen).

Table S1. Relative energies (kcal/mol) of radicals formed via different fragmentation paths calculated at the M06-2X/def2-TZVP and CAM-B3LYP/def2-TZVP levels of theory. Path 2 was consistently the preferred (lowest energy) pathway (black) electronic + thermal (ZPE, rotational, translational) energies (blue) and electronic + thermal Gibbs free enthalpies (red).

No.	ΔE (kcal/mol) (M06-2X/def2-TZVP)					ΔE (kcal/mol) (CAM-B3LYP/def2-TZVP)				
	Path 1	Path 2	Path 3	Path 4	Path 5	Path 1	Path 2	Path 3	Path 4	Path 5
1	16.57	0	16.62	28.64	39.84	17.92	0	17.44	27.58	43.68
	17.96	0	15.16	27.55	39.39	19.59	0	16.04	26.70	43.26
	17.93	0	14.75	26.37	38.57	19.97	0	15.78	25.44	42,75
2	15.21	0	15.19	22.45	38.36	16.62	0	16.08	26.08	42.25
	16.71	0	13.85	26.13	38.03	18.33	0	14.72	24.92	41.88
	16.68	0	13.47	25.17	37.48	18.55	0	14.38	23.96	41.33
3	14.30	0	21.59	26.05	37.38	15.75	0	15.18	25.06	41.30
	15.77	0	20.34	25.09	37.05	17.45	0	13.81	23.88	40.91
	15.88	0	20.15	24.35	36.63	17.90	0	13.64	23.13	40.56
4	14.63	0	14.36	26.05	37.55	15.46	0	14.61	24.30	40.73
	15.48	0	11.70	24.34	36.50	17.78	0	13.90	23.86	41.05
	15.53	0	11.86	23.19	36.01	18.24	0	13.74	23.13	40.71
5	14.04	0	13.74	25.34	36.85	16.05	0	15.23	24.99	41.40
	16.11	0	13.00	25.06	37.22	17.18	0	13.26	23.15	40.36
	16.23	0	12.77	24.12	36.69	17.64	0	13.11	22.45	40.05
6	17.02	0	17.33	28.70	37.19	18.41	0	18.04	26.66	39.41
	18.56	0	15.93	27.60	37.21	20.01	0	16.59	25.42	39.38
	18.80	0	15.70	26.56	36.19	20.36	0	16.22	24.33	38.21
7	15.04	0	15.35	26.53	35.04	16.57	0	16.19	24.59	37.38
	16.74	0	14.07	25.52	35.17	18.27	0	14.83	23.44	37.44
	16.94	0	13.85	24.58	34.15	18.48	0	14.40	22.34	36.28
8	14.14	0	14.42	25.47	34.06	15.71	0	15.30	23.57	36.43
	15.80	0	13.10	24.49	34.19	17.39	0	13.92	22.40	36.47
	16.16	0	12.97	23.76	33.31	17.84	0	13.66	21.51	35.52
9	14.46	0	14.51	25.48	34.23	15.41	0	14.72	22.81	35.86
	15.50	0	12.55	23.74	33.64	17.72	0	14.00	22.38	36.61
	15.79	0	12.34	22.60	32.68	18.17	0	13.76	21.51	35.67
10	13.87	0	13.88	24.76	33.53	16.00	0	15.35	23.50	36.53
	16.14	0	13.22	24.46	34.36	17.12	0	13.36	21.67	35.92
	16.50	0	13.16	23.53	33.37	17.57	0	13.13	20.83	35.00

Table S2. Calculated vertical excitation energies and S–C bond dissociation energies for acrylate and methacrylate derivatives of 2-mercaptobenzothiazole (1-10). The S–C BDE parameters are derived from: electronic energies (black), electronic + thermal (ZPE, rotational, translational) energies (blue) and electronic + thermal Gibbs free enthalpies (red).

No.	S_1 energy (kcal/mol)		T_1 energy (kcal/mol)		BDE[a] (kcal/mol)	
	M06-2X[b]	CAM-B3LYP[c]	M06-2X[b]	CAM-B3LYP[c]	M06-2X[b]	CAM-B3LYP[c]
1	108.76	107.84	80.96	70.67	70.47	62.16
					65.58	57.16
					53.30	44.66
2	112.61	112.03	73.02	72.92	71.99	63.64
					67.01	58.60
					54.98	46.12
3	113.47	112.92	85.18	73.66	73.01	64.64
					68.05	59.61
					55.55	46.95
4	110.03	109.74	84.43	72.75	72.97	65.34
					68.66	59.59
					56.13	46.92
5	110.60	110.16	82.70	71.54	73.67	64.65
					68.09	60.29
					55.56	47.59
6	109.66	108.84	82.58	72.29	70.10	61.69
					65.22	56.74
					52.74	44.29
7	112.58	111.99	73.02	72.92	72.27	63.74
					67.26	58.70
					54.69	46.26
8	113.44	112.88	85.18	78.80	73.29	64.73
					68.29	59.71
					55.50	47.06
9	110.00	109.71	84.43	72.75	73.23	65.42
					68.89	59.67
					56.25	47.02
10	110.57	110.12	82.70	71.54	73.94	64.74
					68.31	60.38
					55.61	47.68

[a] the S–C bond dissociation energies

[b] TD-DFT calculations were performed at the M062X/def2-TZVP level (5 excited states, single-point)

[c] TD-DFT calculations were performed at the CAM-B3LYP/def2-TZVP level (5 excited states, single-point)

Table S3. The values of BDEs for the weakest C–H bonds in the monomers calculated at the M06-2X/def2-TZVP and CAM-B3LYP/def2-TZVP levels of theory (black) electronic + thermal (ZPE, rotational, translational) energies (blue) and electronic + thermal Gibbs free enthalpies (red).

Monomer	BDE (kcal/mol)	
	M06-2X/def2-TZVP	CAM-B3LYP/def2-TZVP
BA	103.39	101.87
	95.17	93.62
	86.88	85.33
HDDA	103.40	101.87
	97.84	96.31
	88.70	87.22
TMPTA	102.43	100.93
	94.42	92.86
	86.09	84.99
HEA	102.37	100.72
	95.23	93.73
	87.05	85.6
PETA	99.75	98.87
	92.08	91.24
	83.90	89.78

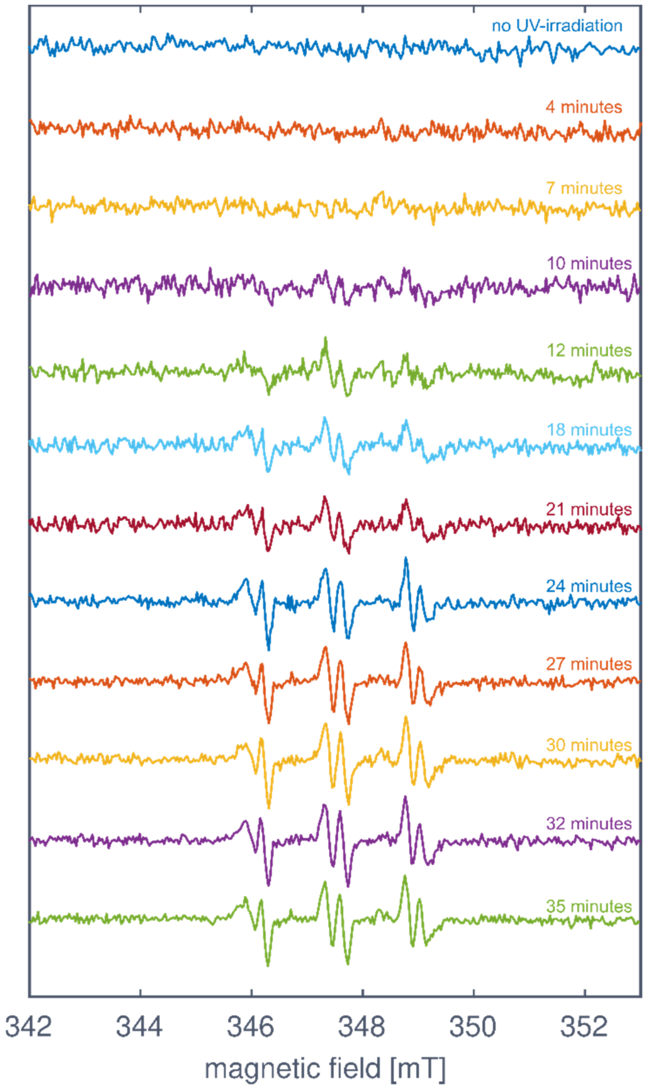

Figure S1. Experimental EPR spectra (common intensity scale) recorded for the *in situ* UV-irradiated sample containing both PBN and photoinitiator 5.

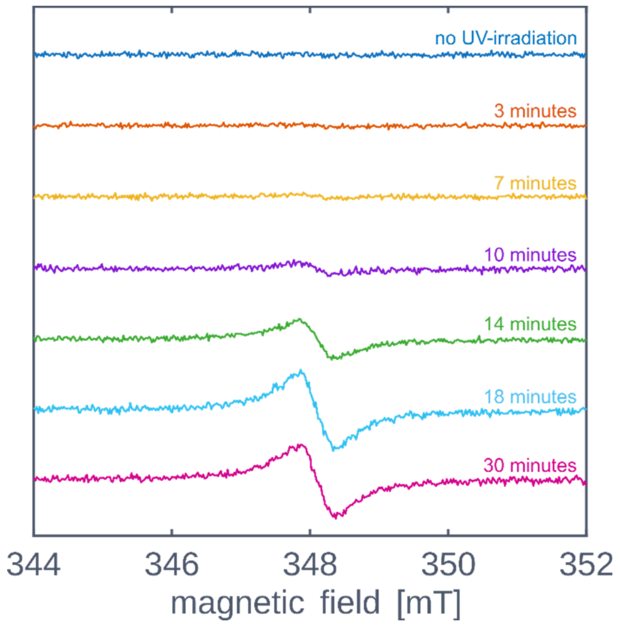

Figure S2. Experimental EPR spectra (common intensity scale) recorded for the *in situ* UV-irradiated sample containing both H_2Q and photoinitiator 5.

Table S4. Computational descriptors used within the correlation study: $S_0 \rightarrow S_1$ and $S_0 \rightarrow T_1$ vertical excitation energies, S–C bond dissociation energies (BDE, given as Gibbs free enthalpies), Mulliken spin populations at the thiyl S (S_S) and thiazole N (S_N) of the thiyl radicals, the spin population sum and difference, together with classical Hammett constants σ of benzothiazole substituents, for acrylate (1-5) and methacrylate (6-10) derivatives of 2-mercaptobenzothiazole. The computational results were obtained at the M06-2X/def2-TZVP level of theory.

Compound	S_1 energy (kcal/mol)	T_1 energy (kcal/mol)	S-C BDE (kcal/mol)	S_S	S_N	S_S+S_N	S_S-S_N	σ [a]
**1**	108.76	80.96	53.30	0.610	0.216	0.826	0.394	-0.24
**2**	112.61	73.02	54.98	0.672	0.195	0.867	0.477	-0.17
**3**	113.47	85.18	55.55	0.700	0.192	0.892	0.508	0.00
**4**	110.03	84.43	56.13	0.689	0.196	0.885	0.493	0.37
**5**	110.60	82.70	55.56	0.713	0.182	0.895	0.531	0.23
**6**	109.66	82.58	52.74	0.608	0.200	0.808	0.408	-0.24
**7**	112.58	73.02	54.69	0.672	0.195	0.867	0.477	-0.17
**8**	113.44	85.18	55.50	0.700	0.192	0.892	0.508	0.00
**9**	110.00	84.43	56.25	0.689	0.196	0.885	0.493	0.37
**10**	110.57	82.70	55.61	0.713	0.182	0.895	0.531	0.23

[a] The Hammett constants were σ_p, with exception of differently substituted compounds 4 and 9, where σ_m was taken instead. The constants are taken from Table 1 of: Hansch, C.; Leo, A.; Taft, R. W. *Chem. Rev.* **1991**, *91*, 165–195.

Table S5. Correlation coefficients between the computational descriptors and the experimental data from Table 4 of the main manuscript (kinetic parameters of radical polymerization of HEA initiated by compounds 1-10; data for C=10% initiator concentration were chosen). Correlation strength is indicated by the color scale from green (weak correlation) to red (strong correlation).

	R_p	$C_{\%}$	$C_{\%}$ after 10 min	$C_{\%}$ after 15 min	$C_{\%}$ after 20 min
S_1 energy	-0.5951	-0.3883	-0.4293	-0.5081	-0.5027
T_1 energy	0.3594	0.0223	0.2742	0.2649	0.1988
S–C BDE	0.0277	-0.1677	-0.0009	-0.0415	-0.1276
S_S	0.0982	-0.0403	0.1790	0.0936	0.0318
S_N	-0.2697	-0.1431	-0.4519	-0.3049	-0.2458
S_S+S_N	0.0388	-0.0951	0.0831	0.0220	-0.0375
S_S-S_N	0.1357	-0.0038	0.2393	0.1391	0.0764
σ	0.4470	0.0906	0.3571	0.3314	0.2563

Table S6. Correlation coefficients between the computational descriptors and the experimental data from Table 6 of the main manuscript (kinetic parameters of radical polymerization of TMPTA initiated by compounds 1-5; data for C=3% initiator concentration were chosen). Correlation strength is indicated by the color scale from green (weak correlation) to red (strong correlation).

	Q_{max}	t_{max}	R_p	I_p	$C_{\%}$
S_1 energy	-0.2902	0.6374	-0.2937	-0.3431	0.1431
T_1 energy	0.2857	-0.2253	0.2820	0.2231	0.5007
S–C BDE	-0.1660	0.2368	-0.1695	-0.07637	0.0082
S_S	0.1544	0.0338	0.1506	0.2149	0.3441
S_N	-0.2831	0.0962	-0.2801	-0.3576	-0.3849
S_S+S_N	0.0954	0.0898	0.0913	0.1487	0.3197
S_S-S_N	0.1857	0.0033	0.1821	0.2499	0.3557
σ	0.0979	-0.1924	0.0955	0.2104	0.0614

Table S7. Correlation coefficients between the computational descriptors and the experimental data from Table 7 of the main manuscript (kinetic parameters of radical polymerization of TMPTA initiated by compounds 6-10; data for C=8% initiator concentration were chosen). Correlation strength is indicated by the color scale from green (weak correlation) to red (strong correlation).

	Q_{max}	t_{max}	R_p	I_p	$C_{\%}$
S_1 energy	-0.4621	0.7085	-0.4599	-0.5844	0.0349
T_1 energy	0.2300	-0.0563	0.2312	0.1872	-0.2937
S–C BDE	-0.6104	0.6838	-0.6095	-0.7125	-0.6031
S_S	-0.3952	0.5734	-0.3936	-0.5594	-0.2601
S_N	-0.2263	0.0216	-0.2278	-0.0428	-0.3237
S_S+S_N	-0.4937	0.6584	-0.4921	-0.6464	-0.3579
S_S-S_N	-0.3155	0.5023	-0.3138	-0.4869	-0.1822
σ	-0.3010	0.2855	-0.3009	-0.3517	-0.5928

Table S8. Correlation coefficients between the computational descriptors and the combined experimental data from Tables 6 and 7 of the main manuscript (kinetic parameters of radical polymerization of TMPTA initiated by compounds 1-10; data for initiator concentration C=3% for compounds 1-5 and 8% for compounds 6-10 were chosen). Correlation strength is indicated by the color scale from green (weak correlation) to red (strong correlation).

	Q_{max}	t_{max}	R_p	I_p	$C_{\%}$
S_1 energy	-0.3300	0.4883	-0.3305	-0.3940	0.1130
T_1 energy	0.2429	-0.1438	0.2424	0.1869	0.1945
S–C BDE	-0.4484	0.3724	-0.4489	-0.4962	-0.2506
S_S	-0.1681	0.1998	-0.1683	-0.2641	0.1027
S_N	-0.2715	0.1675	-0.2708	-0.2170	-0.3966
S_S+S_N	-0.2951	0.3023	-0.2951	-0.3982	0.0048
S_S-S_N	-0.0824	0.1297	-0.0827	-0.1723	0.1654
σ	-0.1315	0.0006	-0.1321	-0.1405	-0.1763

Table S9. Correlation coefficients between the computational descriptors and the experimental data from Table 8 of the main manuscript (kinetic parameters of radical polymerization of PETA initiated by compounds 1-10; data for C=10% initiator concentration were chosen). Correlation strength is indicated by the color scale from green (weak correlation) to red (strong correlation).

	Q_{max}	t_{max}	R_p	I_p	$C_{\%}$
S_1 energy	-0.2864	0.3272	-0.2868	-0.1779	0.3010
T_1 energy	0.1566	0.0331	0.1561	-0.0704	-0.2174
S–C BDE	-0.2038	0.0516	-0.2046	-0.0198	0.0306
S_S	-0.0238	-0.0827	-0.0248	0.1882	0.1866
S_N	-0.2166	0.2543	-0.2158	-0.4078	-0.2975
S_S+S_N	-0.0974	-0.0242	-0.0983	0.1083	0.1407
S_S-S_N	0.0247	-0.1199	0.0238	0.2378	0.2139
σ	0.0341	-0.1712	0.0335	0.1317	-0.0771
